# A higher plant FAD synthetase is fused to an inactivated FAD pyrophosphatase

**DOI:** 10.1016/j.jbc.2022.102626

**Published:** 2022-10-20

**Authors:** Joseph H. Lynch, Sanja Roje

**Affiliations:** Institute of Biological Chemistry, Washington State University, Pullman, Washington, USA

**Keywords:** flavin adenine dinucleotide, FMN, flavin, vitamin, *Arabidopsis*, *Chlamydomonas*, AtFADS1, *Arabidopsis thaliana* FADS1, cDNA, complementary DNA, CgFMNAT, FAD synthetase from *Candida glabrata*, CrFADS1, *Chlamydomonas reinhardtii* FADS1, eGFP, enhanced GFP, FAD, flavin adenine dinucleotide, THP, Tris(hydroxypropyl)phosphine

## Abstract

The riboflavin derivatives FMN and flavin adenine dinucleotide (FAD) are critical cofactors for wide-ranging biological processes across all kingdoms of life. Although it is well established that these flavins can be readily interconverted, in plants, the responsible catalysts and regulatory mechanisms remain poorly understood. Here, we report the cloning and biochemical characterization of an FAD synthetase encoded by the gene *At5g03430*, which we have designated AtFADS1 (*A. thaliana* FADS1). The catalytic properties of the FAD synthetase activity are similar to those reported for other FAD synthetases, except that we observed maximum activity with Zn^2+^ as the associated divalent metal cation. Like human FAD synthetase, AtFADS1 exists as an apparent fusion with an ancestral FAD pyrophosphatase, a feature that is conserved across plants. However, we detected no pyrophosphatase activity with AtFADS1, consistent with an observed loss of a key catalytic residue in higher plant evolutionary history. In contrast, we determined that algal FADS1 retains both FAD synthetase and pyrophosphatase activity. We discuss the implications, including the potential for yet-unstudied biologically relevant noncatalytic functions, and possible evolutionary pressures that have led to the loss of FAD pyrophosphatase activity, yet universal retention of an apparently nonfunctional domain in FADS of land plants.

Flavin adenine dinucleotide (FAD) and FMN are vital to all living organisms because of their function as irreplaceable cofactors for enzymes participating in a wide variety of metabolic processes. These processes include, but are not limited to, mitochondrial electron transport, photosynthesis, fatty acid oxidation, protein folding, chromatin remodeling, and metabolism of several other biologically relevant compounds, such as nucleotides, amino acids, antioxidants, and other cofactors (1, 2, 3, 4, 5). In plants, they also play a significant role in signaling as the chromophores in blue-light receptors (2, 6, 7, 8).

FMN and FAD are derived from riboflavin, for which the *de novo* synthesis pathway in bacteria, yeast, and plants has been characterized and extensively reviewed (5, 9, 10, 11). The phosphorylation of the ribose moiety of riboflavin by riboflavin kinases leads to formation of FMN, whereas FAD is formed *via* the FAD synthase–catalyzed adenylylation of FMN with ATP as the adenylyl donor (10, 12, 13, 14). FAD pyrophosphatase, which cleaves AMP from FAD to form FMN, and FMN hydrolases, which release inorganic phosphate from FMN to form riboflavin, are also involved in the interconversion of these flavins (14, 15, 16, 17, 18, 19).

The enzymes that interconvert flavins are thought to be vitally important to maintaining proper intercompartmental cellular flavin cofactor homeostasis in all organisms, including plants. Previous work utilizing classic biochemical analysis in plants has shown plastids, mitochondria, and the cytosol to each possess their own set of flavin interconverting enzymes (13, 14). However, only a subset of the responsible catalysts have been identified and characterized (5, 13, 14, 19, 20). Two plastidially localized *Arabidopsis thaliana* FAD synthetases, AtRibF1 and AtRibF2, with high homology to each other have previously been reported (14). Two additional flavin-interconverting enzymes from plastids have been characterized: the FMN hydrolase AtcpFHy1 (20) and the FAD pyrophosphatase ATNUDX23 (19, 21, 22). Of the cytosolic enzymes, only the bifunctional enzyme AtFMN/FHy, which possesses riboflavin kinase and FMN hydrolase activities, has been described (13). To date, no additional flavin-interconverting enzymes have been characterized in plants.

Although the *Arabidopsis thaliana* FAD synthetases, AtRibF1 and AtRibF2, are sequence homologs to RibC from *Bacillus subtilis* and RibF from *Escherichia coli* (14), the other eukaryotic FAD synthetases identified to date do not share homology to prokaryotic enzymes (23, 24, 25, 26). Furthermore, while all FAD synthetases described in prokaryotes, with the exception of one from *Methanocaldococcus jannaschii*, are bifunctional, possessing also riboflavin kinase activity (27, 28, 29, 30, 31), eukaryotic FAD synthetases characterized to date all lack the riboflavin kinase activity (14, 16, 23, 24, 25, 26, 32). In addition, the eukaryotic riboflavin kinases characterized to date lack FAD synthetase activity, consistent with a separation of the two activities (13, 33, 34).

A single FAD synthetase, FAD1, has been identified in the model yeast *Saccharomyces cerevisiae* (26). The activity of this essential enzyme (35) is dependent on Mg^2+^ (26). Humans and other animals possess a sequence homolog with a substantial, up to 298 residue, extra domain on the N terminus (36), which was recently identified as an FAD pyrophosphatase (37, 38). The human protein is present as at least two transcript variants, both of which have been shown to also encode Mg^2+^-dependent enzymes, localized in mitochondria and cytosol, respectively (23, 24, 25, 39). For isoform 2 only, it has been shown that Co^2+^ can replace the Mg^2+^ with only a small reduction in activity (40). Several other FAD synthetases have been purified and characterized from a multitude of species, including *M. jannaschii*, *Corynebacterium ammoniagenes*, *B. subtilis*, *Brevibacterium ammoniagenes*, and rat (27, 28, 31, 41). All but one have a functional dependence on Mg^2+^ (28, 31, 41). The sole exception is an archaeal FAD synthetase from *M. jannaschii*, which was reported to have maximum activity with Co^2+^ (27). Since catalytic activity with other metal cofactors is not always tested, the ability of these enzymes to substitute another metal for magnesium cannot be ruled out.

Here, we report the cloning, characterization, and cytosolic localization of an enzyme from *A. thaliana* with sequence homology to the FAD synthetases from animals and yeast. The complementary DNA (cDNA) for this enzyme was cloned and recombinantly expressed. The protein product, named AtFADS1, was purified and characterized as the first FAD synthetase having a strong preference for Zn^2+^ as the associated metal. We also show that the large 243-residue C-terminal domain of the protein, which is homologous to known FAD pyrophosphatases, is not required for the FAD synthetase activity. We also report an apparent lack of FAD pyrophosphatase activity of this enzyme in higher plants, though this activity is present in early diverging lineages, and discuss the implications of such a finding.

## Results

### Plants contain a putative FAD synthetase/FAD pyrophosphatase fusion protein

We previously reported bioinformatic evidence that the gene *At5g03430* from *A. thaliana* encodes a sequence homolog of the *S. cerevisiae* FAD synthetase FAD1 (13). Because of the high homology to *S. cerevisiae* FAD1, we hereafter refer to the protein encoded by gene *At5g03430* as AtFADS1. In addition to the homologous region, the putative *A. thaliana* protein also possesses a substantial, 252-residue, C-terminal extra domain that is homologous to the *S. cerevisiae* FAD pyrophosphatase FPY1 (YMR178w) (Fig. S1). A broader search reveals that FAD1 homologs are conserved across the entire plant kingdom, invariably with the C-terminal fusion to an FPY1-like domain (Fig. S1). As previously reported, human FAD synthetases, hFADS1 and hFADS2, transcript variants encoded by a single gene, are also homologous to *S. cerevisiae* FAD1, and both variants include an extra domain with homology to FPY1, as do homologs in other animal species (36). These contrasting architectures across multiple kingdoms of life are indicative of convergent evolution toward fusion of the two proteins. The presence of two orientations across the different kingdoms reveals two distinct genetic fusion events in evolutionary history, one in an ancestor to humans and other animals, and a separate event in a progenitor to all modern plants, including green algae. Phylogenetic analysis of similar proteins across diverse species demonstrates that the plant proteins form a distinct clade from those in animals, consistent with this proposed evolutionary trajectory (Fig. S2). This convergent evolution is consistent with conserved function of the individual protein domains across the kingdoms, suggesting that AtFADS1 may be a bifunctional FAD synthetase/FAD pyrophosphatase.

### AtFADS1 has FAD synthetase, but not FAD pyrophosphatase, activity

Preliminary attempts at expressing AtFADS1 in bacterial systems were unsuccessful, resulting in formation of inclusion bodies, so instead expression in yeast was pursued. The cDNAs for full-length AtFADS1 and the truncated protein having just the FAD synthetase domain (AtFADS1^trunc^) were cloned by reverse transcription–PCR using mRNA isolated from *A. thaliana* stems as template. Resulting cDNA fragments were subcloned into yeast expression vector pYES-DEST52, followed by functional expression in *S. cerevisiae*. The purified tagged proteins were used in all subsequent work.

**Figure 1:**
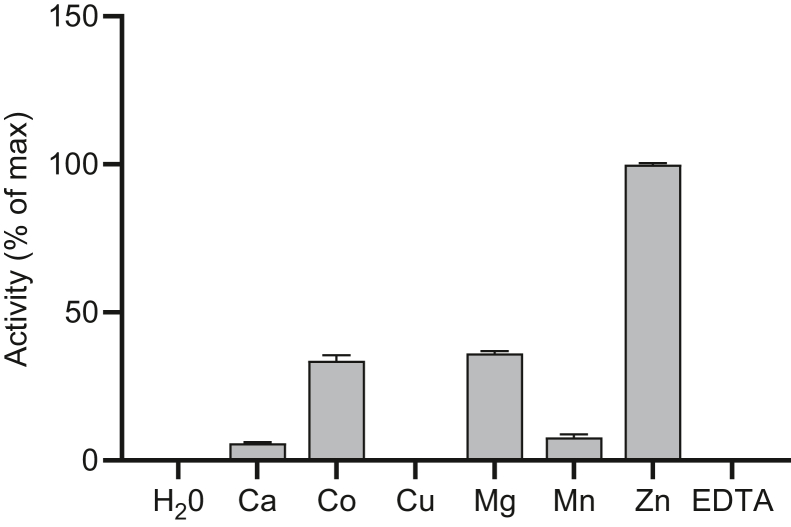
**Metal dependence of AtFADS1.** Recombinant AtFADS1 was assayed for FAD synthetase activity using 15 μM FMN, 10 mM ATP, and 10 mM salt of the divalent metal cations. Metal-free controls were performed using water or 2 mM EDTA in place of the ions. Data are expressed relative to the condition with maximum activity, set to 100%, and are means ± SEM of three replications. AtFADS1, *Arabidopsis thaliana* FADS1.

Recombinant AtFADS1 was assayed for FAD synthetase activity using a variety of metal activators. Consistent with previously characterized FAD synthetases, a divalent metal cation was necessary for activity (Fig. 1). However, while multiple tested metals were capable of sustaining FAD synthetase activity, the enzyme displayed a strong preference for Zn^2+^, which yielded a specific activity threefold higher than any other tested metal (Fig. 1). This marks the first time an FAD synthetase has been characterized with preference for zinc ions for maximum activity.

**Figure 2:**
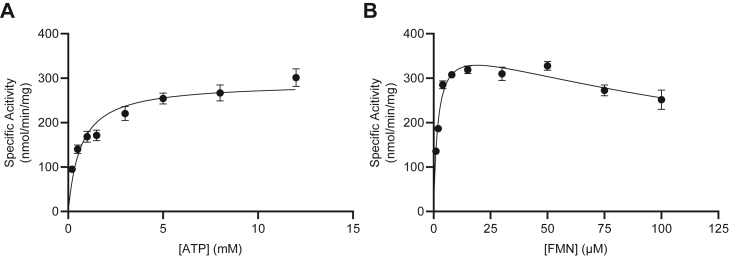
**Steady-state substrate response curves for FAD synthetase activity of AtFADS1.** Initial reaction rates were determined using (*A*) variable ATP concentrations with FMN concentration fixed at 15 μM, or (*B*) variable FMN concentrations with ATP concentration fixed at 10 mM. *Curves* represent a nonlinear best fit to the Michaelis–Menten equation in (*A*) and a model of substrate inhibition for (*B*). Data are means ± SEM of three triplicate determinations. AtFADS1, *Arabidopsis thaliana* FADS1; FAD, flavin adenine dinucleotide.

Substrate response curves of AtFADS1 for both FMN and ATP show Michaelis–Menten kinetics, though with substrate inhibition by FMN (Fig. 2). Substrate inhibition had previously been reported for AtRibF1 and AtRibF2, the plastidial Arabidopsis FAD synthetases that lack homology to AtFADS1 (14), and for FAD synthetase from *C. ammoniagenes* (28), but not from *B. subtilis* (31), rat (41), or human (23, 24, 25). The observed substrate inhibition is consistent with previous proposals that FAD synthetases follow an ordered bi–bi reaction mechanism in which ATP binds prior to FMN (28, 32), as substrates binding out of order would cause inhibition by giving rise to an inactive complex.

The kinetic parameters of this enzyme reveal that AtFADS1 has a *K*_*m*_ for FMN of 1.9 μM (Table 1). This is a similar affinity as reported for homologous enzymes from other species, though it is somewhat lower than for nonhomologous enzymes from the same species, AtRibF1 and AtRibF2 (14). The *K*_*m*_ for ATP (665 μM) is approximate two-fold to 60-fold higher for AtFADS1 than the 11 to 300 μM reported for other characterized FAD synthetase isoforms from multiple source organisms (14, 28, 31, 41). Some of these differences may be attributed to differences in reaction conditions, including buffer composition, pH, and temperature.

**Table 1:** Kinetic parameters determined in this study

Enzyme	Substrate varied	*K*_*m*_ (μM)	*K*_cat_ (s^−1^)	*K*_cat_/*K*_*m*_ (s^−1^ mM^−1^)	*K*_*i*_ (μM)
AtFADS1^a^	ATP	665 ± 11	0.287 ± 0.011	0.432 ± 0.018	NA
AtFADS1^a^	FMN	1.91 ± 0.25	0.393 ± 0.017	206 ± 28	190 ± 36
AtFADS1^trunc1^	FMN	10.0 ± 5.5	0.695 ± 0.236	69.5 ± 44.9	32.7 ± 18.2
CrFADS1^a^	FMN	0.54 ± 0.09	0.103 ± 0.004	191 ± 33	336 ± 66
CrFADS1^b^	FAD	4.88 ± 0.56	0.395 ± 0.011	81.0 ± 9.6	NA

Abbreviation: NA, not applicable.

a Assayed for FAD synthetase activity.

b Assayed for FAD pyrophosphatase activity.

AtFADS1 is active over a broad range of neutral pH values (Fig. S4). This would support activity in the proposed localization in the cytosol, an environment that is typically neutral to slightly basic. Similarly, the FAD synthetase isolated from rat and the human FAD synthetase isoform 2 both have a maximum activity at neutral pH values (40, 41).

Assays revealed that the truncated AtFADS1^trunc^ protein lacking the FPY1-like domain also possessed FAD synthetase activity. As with the full-length AtFADS1, the truncated protein displayed highest activity in the presence of Zn^2+^ among all tested metal ions (Fig. S5). AtFADS1^trunc^ exhibited a higher *V*_max_ than the full-length protein that can be partially attributed to the decrease in molecular weight; however, *K*_*m*_ for FMN was also increased in the truncated protein, leading to a net decrease in catalytic efficiency (*K*_cat_/*K*_*m*_) (Table 1 and Fig. S5). Therefore, we conclude that the FPY1-like domain is not directly involved in FAD synthetase catalytic function.

To test whether the FPY1-like domain might retain the same enzymatic function as its yeast homolog, the full-length AtFADS1 was assayed for FAD pyrophosphatase activity. None was detected despite the tests that utilized all possible combinations of various pH buffers at 100 mM (Tris, phosphate, Mops, Hepes, tricine, and imidazole), 10 mM divalent metal cations (Mg^2+^, Mn^2+^, Co^2+^, Zn^2+^, Ni^2+^, Ca^2+^, Cu^2+^, and Fe^2+^), and 200 mM salts of monovalent cations (KCl and NaCl). Attempts using other additives (10 mM Na_2_B_4_O_7_, 10% glycerol, 10 mM NH_4_SO_4_, 0.7 M urea, 100 mM LiCl_2_, and 1 mM (NH_4_)_2_MoO_4_) also failed to give rise to any FAD pyrophosphatase activity. Previous analysis of this family of pyrophosphatase-like enzymes identified critical residues for catalysis, including an Asp residue involved in coordinating the catalytic metal ion. This residue corresponds to the Asn at position 296 of AtFADS1 (Fig. S1) (42). The importance of this residue has been demonstrated experimentally with a homolog from *Agrobacterium tumefaciens*, as mutation of the Asp to an AtFADS1-like Asn resulted in complete loss of activity (42). Therefore, this substitution, which results in loss of charge and therefore inability to coordinate the metal cation, likely explains the lack of FAD pyrophosphatase activity despite sequence homology to known FAD pyrophosphatases.

### Algal FADS1 retains FAD pyrophosphatase activity

**Figure 3:**
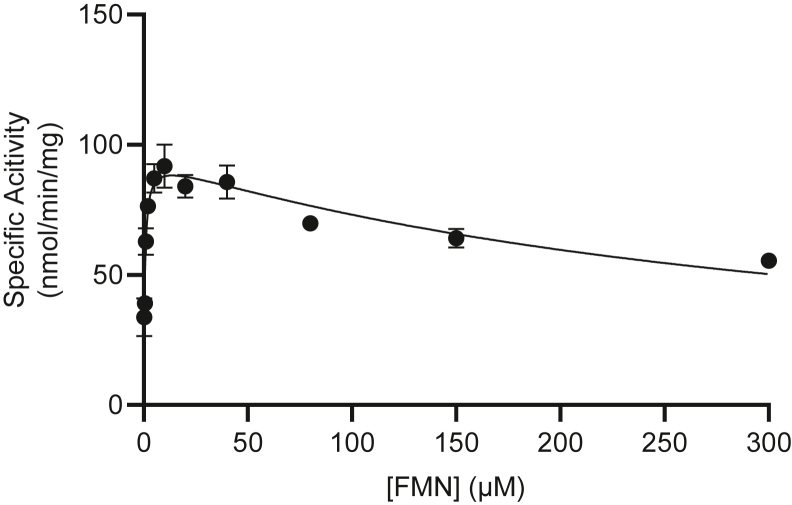
**Steady-state substrate response curve for FAD synthetase activity of CrFADS1.** Initial reaction rates were determined using variable FMN concentrations with ATP concentration fixed at 10 mM. *Curves* represent a nonlinear best fit to a model of substrate inhibition. Data are means ± SEM of three triplicate determinations. CrFADS1, *Chlamydomonas reinhardtii* FADS1; FAD, flavin adenine dinucleotide.

The aforementioned lack of a critical active-site residue in AtFADS1 is a conserved feature of land plant FADS1 homologs, but not those of green algae (Fig. S1), the latter of which form a clade with the *S. cerevisiae* FPY1 in phylogenetic analysis (Fig. S2). Thus, we hypothesize that algae, in contrast to their terrestrial relatives, possess bifunctional FADS1 enzymes capable of catalyzing both the formation and degradation of the FAD cofactor. To test this, we produced recombinant *Chlamydomonas reinhardtii* FADS1 (CrFADS1) under the same conditions as used for AtFADS1. As expected, CrFADS1 exhibits FAD synthetase activity under the same conditions as were found to be optimal for AtFADS1 (Fig. 3). Kinetic parameters of CrFADS1, including substrate inhibition, are similar to those observed for AtFADS1 (Table 1), consistent with conserved function despite the substantial divergence between the two species.

**Figure 4:**
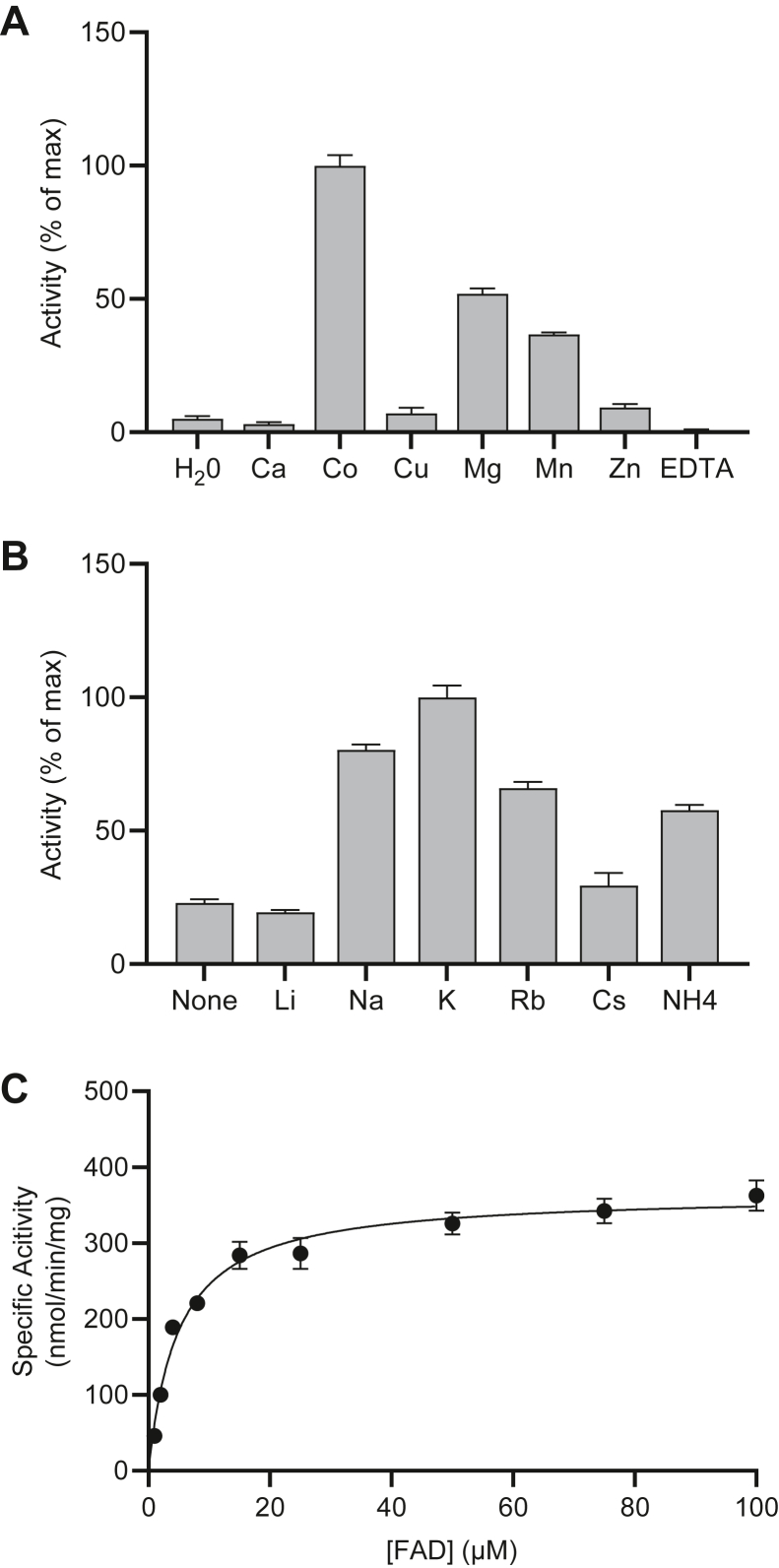
**FAD pyrophosphatase activity of CrFADS1.***A*, FAD pyrophosphatase activity of recombinant CrFADS1 was determined in the presence of 5 mM salt of the divalent metal cations listed. Metal-free controls were performed using water or 2 mM EDTA in place of the ions. *B*, CrFADS1 was assayed for FAD pyrophosphatase activity in the presence of 200 mM chloride salt of the listed monovalent cations. *C*, initial reaction rates for the FAD pyrophosphatase activity of CrFADS1 were determined using variable FAD concentrations in the presence of 2 mM CoCl_2_ and 200 mM KCl. *Curves* represent a nonlinear best fit to a model of substrate inhibition. All data are means ± SEM of three triplicate determinations. CrFADS1, *Chlamydomonas reinhardtii* FADS1; FAD, flavin adenine dinucleotide.

As we hypothesized based on analysis of the primary structure, CrFADS1 also exhibits FAD pyrophosphatase activity. As reported for *S. cerevisiae* FPY1 (36), the activity is highest in the presence of potassium chloride and Co^2+^ (Figs. 4, *A* and *B* and S6). Unlike FPY1, chloride salts of most other group 1 cations tested, except lithium, as well as ammonium chloride could also promote FAD pyrophosphatase activity (Fig. 4*B*), and therefore, it is likely that the low activity in the absence of any exogenously provided salt is due to background levels of Na^+^ present in the reaction buffer. As reported for FPY1, Mg^2+^ could replace the Co^2+^, although specific activity was lower with the former, even at the higher optimal concentration (Figs. 4*A* and S7). Substrate response curves display typical Michaelis–Menten kinetics (Fig. 4*C*).

### Modeled AtFADS1 pyrophosphatase-like active site reveals noncatalytic flavin binding

To better understand the structural basis of the differential results obtained with the algal and Arabidopsis FADS1 proteins in FAD pyrophosphatase assays, we undertook a structural modeling approach. Previously, the crystal structure had been determined for *Thermus thermophilus* CinA protein in complex with its substrate ADP-ribose and Mg^2+^ (43). As CinA is 23.3% identical to AtFADS1 across the two proteins’ respective pyrophosphatase domains, its quaternary structure was used as a template for homology-based modeling of AtFADS1 structure using SWISS-MODEL webtool (Swiss Institute of Bioinformatives) (44). Overlaying the modeled AtFADS1 structure with the CinA structure confirmed, as reported previously (42) and described previously, that a metal-coordinating negatively charged Asp (Asp45 in CinA) is replaced by a neutral Asn at position 296 in AtFADS1 and in the corresponding location in related FADS1 proteins of higher plants (Figs. S1 and S3*A*). An adjacent Asp (Asp297) is unable to substitute because of being constrained in an opposing orientation by an alpha helix (Fig. S3*A*). Further examination of the active site revealed Arg458 of AtFADS1 conspicuously protruding into the substrate-binding pocket from the equivalent location of CinA Pro210 (Fig. S3*B*). However, the position of the Arg side chain, the presence of which is perfectly anticorrelated with retention of the metal-coordinating Asp among analyzed sequences (Fig. S1), does not block substrate in the bound configuration but instead favorably positions the positive amine charge near the expected location of the negatively charged pyrophosphate moiety of FAD (Fig. S3*C*). Thus, we conclude that the pyrophosphatase-like domain of FADS1 proteins of higher plants may have evolved to promote noncatalytic stable binding of FAD in the absence of the ancestral catalytic metal cation.

### GFP-fused AtFADS1 localizes to the cytosol in *A. thaliana* protoplasts

**Figure 5:**
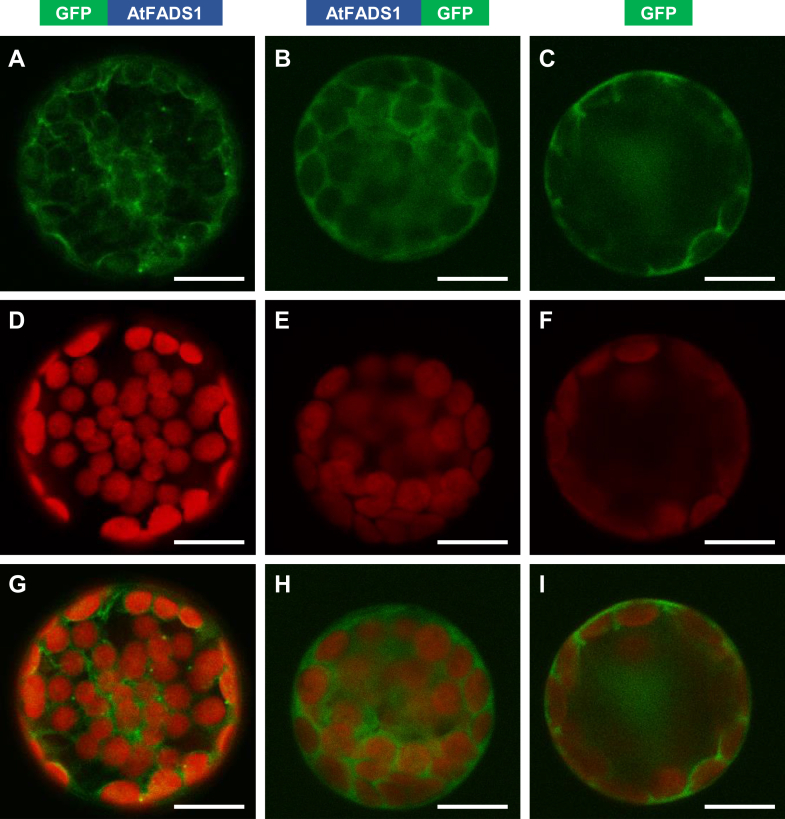
**Subcellular localization of AtFADS1.** Arabidopsis protoplasts transiently expressing full-length AtFADS1 with either an N-terminal (*A*, *D*, and *G*) or C-terminal (*B*, *E*, and *H*) eGFP fusion were observed by confocal microscopy. Nonfused eGFP was used as a cytosolic control (*C*, *F*, and *I*). *A*–*C* show eGFP fluorescence; *D*–*F* show chlorophyll autofluorescence; *G*–*I* show merged images. The scale bars represent 10 μm. AtFADS1, *Arabidopsis thaliana* FADS1; eGFP, enhanced GFP.

Subcellular localization of AtFADS1 was studied through transient expression of the full-length protein with an N-terminal or a C-terminal enhanced GFP (eGFP) fusion in *A. thaliana* protoplasts. Expression of either fusion protein resulted in green fluorescence being visible throughout the cytosol, nearly identical to the results seen with control protoplasts expressing eGFP alone (Fig. 5). Neither was there colocalization of green fluorescence with the red autofluorescence of the chloroplasts nor was there any accumulation of green fluorescence indicative of protein localization to any subcellular structure. Therefore, these findings reveal that AtFADS1 is a cytosolic protein.

## Discussion

Flavin cofactors are required for a variety of metabolic processes in plastids, mitochondria, and cytosol. Our laboratory has previously reported evidence for the presence of flavin interconverting enzymes in each of these subcellular compartments (14). However, many of these enzymes have yet to be identified, even though their identification and characterization is critical to understanding flavin cofactor biosynthesis. In this study, we report the cloning, recombinant expression, and characterization of AtFADS1, an FAD synthetase from *A. thaliana* that localizes to the cytosol.

Analysis of the amino acid sequence demonstrates high homology between the first 235 residues of this enzyme and the entire sequence of a previously functionally characterized *S. cerevisiae* FAD synthetase. Residues 235 through the end (497) have high homology to a separate protein (FPY1) encoded by the *S. cerevisiae* genome, which has recently been shown to be an FAD pyrophosphatase (36). This apparent fusion seen in *A. thaliana* is widespread across plant species, observed not only in vascular plants but also in green algae as well (Fig. 1). We also observed that homologs of the two *S. cerevisiae* proteins, FAD1 and FPY1, are expressed as fusion proteins in a wide variety of animal species, except the orientation of the two domains is reversed relative to plants (Fig. S1), indicating that this combination arose from a separate evolutionary fusion event. It was recently reported that both the FAD synthetase and FAD pyrophosphatase activities are maintained in the human enzyme, with the latter activity being specific to the FPY1-like domain (37, 38). In contrast, our experiments with AtFADS1 suggest that it is solely an FAD synthetase.

Recent work demonstrated that among the broader family of pyrophosphatase-like enzymes with sequence homology to this protein, there are five amino acid residues that are conserved across all species except vascular plants (42). This includes one aspartate, replaced by asparagine at position 296 in Arabidopsis, which was shown by mutagenesis to be critical to enzymatic activity in a prokaryotic sequence homolog. To test whether ancestral plant FADS1 homologs with preserved presumptive critical residues might retain the pyrophosphatase functionality, we tested recombinant CrFADS1 for this activity. As hypothesized, CrFADS1 is bifunctional, having both FAD synthetase and pyrophosphatase activities. Based on the totality of evidence, we conclude that during evolution history of plants, fusion between the homologs of yeast FAD1 and FPY1 occurred, whereas both domains retained ancestral enzymatic function. However, following the divergence between modern day green algae and vascular plants, mutation of the pyrophosphatase domain in an early ancestor to vascular plants led to loss of FAD-degrading activity in that lineage, though surprisingly, there is universal retention of the catalytically inactive domain.

The loss of one of the activities in a bifunctional enzyme in flavin cofactor metabolism is a common occurrence in plant evolution. The other two FAD synthetases characterized from plants, AtRibF1 and AtRibF2, are homologous to a full-length prokaryotic bifunctional riboflavin kinase/FAD synthetase, yet neither Arabidopsis protein has riboflavin kinase activity (14). Likewise, in *E. coli*, two consecutive steps of the riboflavin biosynthetic pathway, catalyzed by pyrimidine deaminase and pyrimidine reductase, are carried out by a single bifunctional protein, RibD (45). However, the Arabidopsis genome has been shown to encode two full-length homologs of RibD: the deaminase PyrD and the reductase PyrR (45, 46). Similar to AtFADS1, which has a pyrophosphatase-like domain that lacks catalytic residues, PyrD has a reductase-like domain lacking catalytic residues, and PyrR has a deaminase-like domain that lacks catalytic residues (46). Therefore, all five of these Arabidopsis enzymes have retained an apparently nonfunctional domain. Speculation on potential reasons behind this trend in Arabidopsis leads to several possible explanations: (1) the inactive domains are essential for *in vivo* protein–protein interactions, as proposed previously (46); (2) while lacking catalytic function, the inactive domains may maintain flavin-binding ability and therefore lend themselves to regulation of flavin metabolism or, as posited previously for a human enzyme, chaperoning flavin compounds to other proteins (40); or (3) it is possible that one or more of these enzymes does maintain its ancestral activity, and the experimental methods have failed to create the proper conditions. Further studies will be needed to address these possibilities on an enzyme-by-enzyme basis. We view explanation 3 as unlikely for AtFADS1, because of the apparent loss of a residue necessary for coordinating the catalytic metal cation. On the other hand, explanation 2 warrants further study in light of the potential for noncatalytic binding of FAD suggested by the structural model, especially as a regulatory role for the noncatalytic domain would be consistent with the differing FAD synthetase kinetics observed with AtFADS1 *versus* AtFADS1^trunc^ (Table 1).

The *in vitro* characterization of AtFADS1 revealed a preference for Zn^2+^ for maximum FAD synthetase activity. This was unexpected since all other known FAD synthetases have been reported as preferring Mg^2+^, with the single exception of a Co^2+^-dependent archaeal FAD synthetase. Previous crystal structure determination of CgFMNAT, a FAD synthetase from *Candida glabrata*, identified five amino acids involved in coordinating octahedral Mg^2+^ in that enzyme (47), corresponding to the strictly conserved Asp47, Gly133, Asp138, Ser192, and Arg229 residues of AtFADS1 (Fig. S1). It is possible that other alterations to the active site may induce subtle changes to facilitate binding of the slightly larger Zn^2+^ ion. For example, overlaying a modeled AtFADS1 structure with CgFMNAT reveals that Ile91, Phe131, Leu132, Val134, Gly137, Gln144, and Ile163 of AtFADS1, residues that surround the metal-binding region, replace Leu121, Val165, Ile166, Ile168, Thr171, Leu178, and Leu197, respectively, of CgFMNAT (Fig. S8). These substitutions do not drastically alter the side-chain properties, with the exception of L178Q (CgFMNAT numbering), which is 12 Å from the metal ion in the model and thus too far to be directly involved in its coordination. Indeed, isoform 2 of the human FAD synthetase retains conserved identity with yeast FAD synthetases at four of the seven noted residues (36), and while Co^2+^ can replace Mg^2+^ in assays with nearly no loss in activity, Zn^2+^ nearly eliminates catalytic function of the human enzyme (40). The physiological consequences of a Zn^2+^-dependent FAD synthetase are not clear. It is known that total intracellular Zn^2+^ concentrations for *A. thaliana* can vary by more than an order of magnitude, depending on environmental conditions (48), though concentrations within any particular subcellular compartment have not been determined. If this dramatic fluctuation indeed reflects cytosolic variations of Zn^2+^ concentrations across a range relative to the affinity of AtFADS1, the reliance on zinc ions for efficient FAD synthesis could cause FAD-dependent processes to be dependent on the environmental trace element composition.

A number of previous studies purified, identified, and characterized FAD synthetases, adding significant information to this field of research (14, 23, 24, 25, 26, 27, 30, 31, 41). Several of those studies addressed the catalytic characteristics of the enzymes only when using Mg^2+^ as the associated metal (14, 23, 24, 25, 26, 28). Our discovery of a Zn^2+^-dependent FAD synthetase points to the possibility that these enzymes may be able to use, or even prefer to use, other divalent metal ions for catalysis. Such a possibility warrants further study.

**Figure 6:**
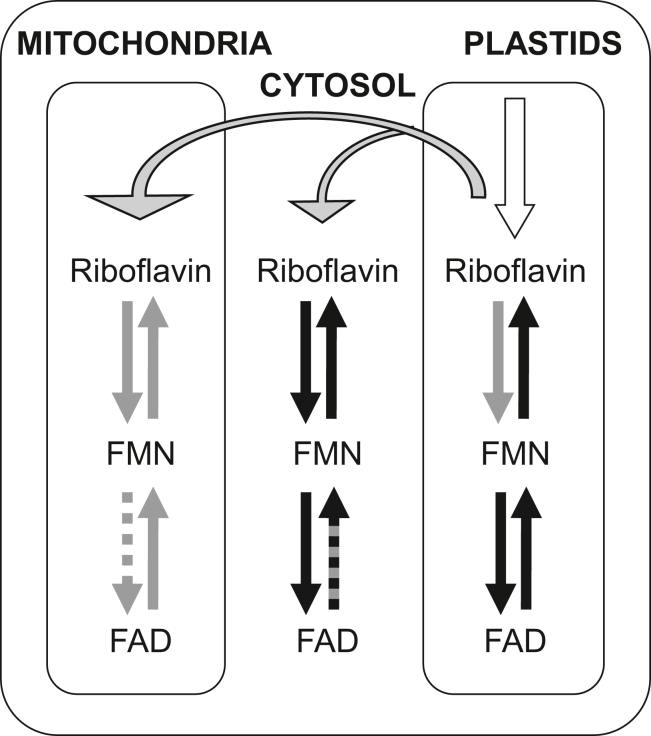
**Proposed model of flavin cofactor metabolism in plants.***Open arrow* represents *de novo* riboflavin biosynthesis. *Solid black arrows* represent identified enzymes. *Solid gray arrows* represent steps for which there is experimental evidence, but the responsible catalyst has not yet been identified. The *dashed gray arrow* represents an enzyme proposed to be present, but for which there is no experimental support. The *black/gray dual color arrow* represents a step supported by experimental evidence, but the identified catalyst is not universally present in plants.

The presented results, as well as recent results from other research groups (9, 19, 21, 22, 46), allow us to update the previously proposed model of flavin cofactor metabolism in plants (Fig. 6). Riboflavin synthesis takes place in plastids (5, 9, 46, 49). Experimental evidence supports FMN synthesis in mitochondria and plastids, as well as FMN hydrolysis in mitochondria, by as yet unidentified enzymes (14). Enzymes responsible for cytosolic FMN synthesis and hydrolysis (AtFMN/FHy) and plastidial FMN hydrolysis (AtcpFHy1) have been identified, although their function may be redundant with other unidentified enzymes (13, 20). Synthesis of FAD from FMN is catalyzed in the cytosol by AtFADS1 (this work) and in the plastids by AtRibF1 and AtRibF2 (14). Though AtFADS1 is incapable of hydrolyzing FAD to FMN (this work), related enzymes in algae are functional, and the activity has been detected in cytosolic preparations from other higher plants suggesting this function is carried out by unidentified enzymes (50). The Nudix protein AtNUDX23 acts as an FAD pyrophosphatase in the plastids (19, 21, 22). The mitochondria have been shown to possess FAD pyrophosphatase activity, although the catalysts have yet to be identified (14). Neither have enzymes been identified that would meet the extensive need for FAD synthesis in the mitochondria, nor are we aware of any reports of direct detection of FAD synthesis in mitochondrial extracts. However, experiments using *Nicotiana tabacum* revealed that intact mitochondria are capable of synthesizing FAD from exogenously supplied riboflavin (51). As each of the subcellular compartments possesses its own set of flavin interconverting enzymes, we hypothesize the riboflavin to be transported across the organellar membranes *via* as yet unidentified mechanisms.

## Experimental procedures

### Materials

Except as otherwise noted, all chemicals and custom PCR primers were obtained from Sigma. YeastBuster reagent was from Novagen. Yeast -Ura dropout supplement was from Clonetech. FMN and FAD were from Sigma and in addition purified as described previously (14).

### *In silico* analysis

Comparison of putative FAD synthetases from various species was conducted as follows. Using the BLASTp suite available from the National Center for Biotechnology Information (http://www.ncbi.nlm.nih.gov/), with At5g03430 as the query sequence, FAD synthetase homologs were identified in multiple species. For further analysis, we selected the homologs from two additional dicots (*Glycine max*, XP_003554393.1 and *N. tabacum*, XP_016463627.1), two monocots (*Oryza sativa*, XP_015621933.1 and *Zea mays*, AQK89528.1), a moss (*Sphagnum magellanicum*, KAH9570159.1), a fern (*Ceratopteris richardii*, KAH7289897.1), a liverwort (*Marchantia polymorpha*, PTQ37944.1), and two green alga (*C. reinhardtii*, XP_001693086.1 and *Chlorella sorokiniana*, PRW59554.1). Alignment of these sequences with *S. cerevisiae* FAD1 and FPY1 was performed using Clustal Omega webtool (https://www.ebi.ac.uk/Tools/msa/clustalo/) (52). For phylogenetic analysis, additional plant sequences were extracted from the Phytozome database (53), and animal sequences were as described previously (36). Full sequences are provided in Dataset S1. The phylogeny was constructed in MEGA, version 11.0.13 (54), using the neighbor-joining method under the default settings.

Homology-based modeling was performed using the SWISS-MODEL webtool (44). The experimentally determined crystal structure of the *C. glabrata* protein CgFMNAT cocrystalized with FAD and Mg^2+^ (Protein Data Bank code: 3G6K) was selected as template for the FAD synthetase domain of AtFADS1 (47). The experimentally determined crystal structure of *T. thermophilus* CinA protein cocrystalized with ADP-ribose and Mg^2+^ (Protein Data Bank code: 4UUX) was selected as template for the pyrophosphatase-like domain of AtFADS1 (43). Structures were visualized using PyMOL Molecular Graphics System, version 2.5.2 (Schrödinger, LLC).

### cDNA cloning, constructs, and sequence analysis

To clone the AtFADS1 cDNA, total RNA was isolated from *A. thaliana* stems using the RNeasy Plant Mini Kit (Qiagen) and reverse-transcribed using Superscript II reverse transcriptase (Invitrogen) and an oligo(dT) primer. The AtFADS1 ORF was then amplified using Taq2000 polymerase (Stratagene) and the primers 5′-GACGACAAGATGGAGATCGATAAAGCGAT-3′ (FADSForward) and 5′-GAGGAGAAGCCCGGTCTACTTGATTTCTACGAACA-3′ (FADSReverse). The generated PCR fragment was purified using a Wizard PCR column (Promega) and cloned into the pGEM-T Easy vector (Promega). The AtFADS1 ORF, excluding the native stop codon, and the truncated AtFADS1^trunc^ ORF, were amplified using *PfuTurbo* DNA polymerase (Stratagene) and the primer pairs 5′-AAAAAGCACGCTATGGAGATCGATAAAGC-3′ (forward for both amplicons) and either 5′-AGAAAGCTGGGTCCTTGATTTCTACGAACAC-3′ (reverse for AtFADS1) or 5′-AGAAAGCTGGGTCCAAAAGCACCTCGTGTTT-3′ (reverse for AtFAD). Note that the primers consist of sequence-specific regions (plain text) and of sequences needed for the further downstream amplification by *attB* primers (underlined). The resulting PCR fragments were reamplified using *attB* primers (Invitrogen) and then separated by agarose gel electrophoresis and purified using Wizard PCR columns (Promega), before subcloning by BP recombination into the pDONR221 vector (Invitrogen). The coding sequence for CrFADS1 was synthesized with flanking Gateway *attL* sites by Genscript. The ORFs for all three enzymes were introduced into the pYES-DEST52 destination vector from the pDONR221 constructs *via* LR recombination.

For expression of AtFADS1 in plants with a C-terminal eGFP fusion, the pDONR221 construct described previously was used to introduce the AtFADS1 ORF into the p2GWF7 vector (55) *via* LR recombination. For expression of AtFADS1 in plants with an N-terminal eGFP fusion, cloning was carried out as for the C-terminal fusion construct, except amplification of full-length AtFADS1 was carried out using the primer pair 5′-AAAAAGCACGCTTCATGGAGATCGATAAAGC-3′ and 5′-AGAAAGCTGGGTCTACTTGATTTCTACGA-3′, and the final destination vector was p2FGW7 (55). All cloning procedures were done in accordance with the manufacturer’s protocols. The constructs were verified by DNA sequencing.

### Expression in *S. cerevisiae*

*S. cerevisiae* strain Y258 from Open Biosystems was used with the S.c. EasyComp Transformation Kit (Invitrogen) for generating chemically competent yeast, and the expression vectors were transformed into these yeasts following the manufacturer’s protocol. Yeasts carrying the expression vectors were cultured at 30 °C in synthetic -Ura dropout medium (0.67% yeast nitrogen base with ammonium sulfate, 0.077% -Ura dropout supplement, and 2% raffinose). Once absorbance reached 1.0 to 1.2 at 600 nm, 50% of the culture volume of 3× expression medium (3% yeast extract, 6% tryptone, and 6% galactose) was added, and incubation was continued for 6 h before harvesting cells by centrifugation at 5000*g* and 4 °C for 10 min.

### Recombinant protein isolation

Extracts of the *S. cerevisiae* cells expressing the recombinant proteins were prepared by resuspending the pellets in 1× YeastBuster with supplied Tris(hydroxypropyl)phosphine (THP) solution at a ratio of 5 ml of YeastBuster per 1 g wet weight of cell pellet. After incubating at room temperature for 20 min with gentle shaking, the extracts were cleared by centrifugation at 16,000*g*, 4 °C for 20 min. The recombinant proteins were purified as follows from the cell lysate using an Äkta FPLC system equipped with 1 ml immobilized metal affinity chromatography columns (GE Healthcare) charged according to the manufacturer’s protocol: a column charged with Cu^2+^ was equilibrated with binding buffer (50 mM potassium phosphate, pH 8.0, 500 mM NaCl, 20 mM imidazole, 1 mM THP, and 0.5% Tween-20) before directly loading the clarified cell lysates in YeastBuster solution. Unbound proteins were removed by washing with 15 column volumes of binding buffer, followed by elution of bound proteins by a linear gradient of binding buffer to elution buffer (50 mM potassium phosphate, pH 8.0, 500 mM NaCl, 500 mM imidazole, 1 mM THP, and 0.5% Tween-20) over 15 column volumes. Target proteins eluted at about 125 mM imidazole. Fractions containing the desired protein were pooled and loaded directly onto an Ni^2+^-charged immobilized metal affinity chromatography column equilibrated with start buffer, which was then washed with 10 column volumes of binding buffer before elution with a linear gradient of binding buffer to elution buffer over 20 column volumes. Target proteins eluted at about 225 mM imidazole. Fractions containing the desired protein were pooled and immediately desalted using Zeba Desalt Spin Columns (Thermo Scientific) into an optimized storage buffer (50 mM potassium phosphate, pH 8.0, 500 mM l-arginine, 1 mM THP, and 0.5% Tween-20). Desalted proteins were aliquoted and stored at −80 °C until use.

### Enzyme assays

Unless otherwise indicated, the procedures described later were used. Initial reaction rates at steady state were measured. Product formation was proportional to enzyme concentration and time. Less than 10% of the substrates were consumed in reactions. For FAD pyrophosphatase assays, unless otherwise noted, the reaction mixture consisted of 15 μM FMN, 10 mM ATP, 100 mM Hepes–NaOH, pH 7.0, and 1 mM THP. Zinc acetate was added to a final concentration of 2 mM higher than the concentration of ATP in the assay, per previous recommendations for assays requiring ATP and a metal ion (56). For FAD pyrophosphatase assays, the reaction mixture consisted of 30 μM FAD, 100 mM Hepes–NaOH, pH 7.0, 200 mM KCl, 2 mM CoCl_2_, and 1 mM THP, unless otherwise noted. Assay volume was 25 μl.

Reactions were incubated at 30 °C for 25 min and then quenched by addition of saturated formic acid to 5% of assay volume. Precipitated protein was removed by centrifugation at 1500*g* for 15 min at 4 °C. Reaction products and substrates were separated by reversed-phase chromatography using an Alliance 2695 HPLC system with a 2475 fluorescence detector (Waters) linked to a Waters SunFire C_18_ column (4.6 × 150 mm, 3.5 μm) and were measured by fluorescence detection using an excitation wavelength of 470 nm and an emission wavelength of 530 nm. The mobile phase contained 100 mM ammonium formate, 100 mM formic acid, and 25% methanol. Product formation was determined from fluorescence by comparison to standards after subtraction of a blank in which the assay was quenched after 5 min to account for reaction progress during handling. FMN and FAD concentrations in standard solutions were determined spectrophotometrically (57).

The *K*_*m*_ and *K*_*i*_ for FMN, as well as the enzyme *V*_max_, were determined by varying concentration of one substrate at a time and holding all other parameter constant. The results were fit by nonlinear best fit to either the standard Michaelis–Menten equation or, if substrate inhibition was apparent, a model of substrate inhibition using the default parameters of GraphPad Prism, version 9.4.0 (GraphPad Software, Inc).

To test for metal dependence, assays were completed as described previously, except MgCl_2_, MnCl_2_, Cu(II) SO_4_, CaCl_2_, CoCl_2_, or 2 mM EDTA, were substituted for Zn CH_3_CO_2_^−^ in the reaction mixture.

### Transient expression of eGFP-fused proteins in *A. thaliana* protoplasts

*A. thaliana* protoplasts were isolated from the leaves of 4-week-old plants and transformed with the plasmid constructs described previously for P35S promoter-driven expression of AtFADS1 with C-terminal or N-terminal eGFP fusions or a positive control in which eGFP was expressed along, using published methods (58). Fluorescence was monitored using a TCS SP5 confocal laser-scanning microscope (Leica Microsystems). eGFP fluorescence was excited at 488 nm and measured at 505 to 530 nm. Chlorophyll fluorescence was excited at 488 nm and measured at 650 to 730 nm.

## Data availability

All data used are provided in this article and the accompanying supporting information.

## Supporting information

This article contains supporting information.

## Conflict of interest

The authors declare that they have no conflicts of interest with the contents of this article.
