# Supplementary material for: A higher plant FAD synthetase is fused to an inactivated FAD pyrophosphatase
Source: J Biol Chem. 2022 Oct 20;298(12):102626. doi: 10.1016/j.jbc.2022.102626 (PMC9678776; doi:10.1016/j.jbc.2022.102626)
Supplement: Supplemental Dataset 1 [file mmc1.pdf]

>A.lyrata v2.1|AL6G12780.t1
MEIDKAIGESDDKRLKTKYNNAIFVIKRALALYSIEEVAFSFNGGKDSTVLLHLLRAGYFLHKKEQTCSN
GGLSSFPVRTIYFESPSAFTEINAFTYDAAQTYNLQLDIIRQDFKSGLEALLKANPIRAIFLGVRIGDPT
AVGQEQFSPSSPGWPPFMRVNPILDWSYRDVWAFLLTCKVKYCSLYDQGYTSIGSIHDTVPNSLLSVNDN
SSKEKFKPAYLLSDGRLERAGRVKKIASLKYDVETESQKHEVLLASVIAVGDEILSGTVEDQLGLSLCKK
LTSVGWSVQQTTVLRNDIDSVSEEVDRQRSTSDMVFIYGGVGPLHSDVTLAGVAKAFGVRLAPDEEFEEY
LRHLISEQCTGDRNEMAQLPEGITELLHHEKLSVPLIKCRNVIVLAATNTEELEKEWECLTELTKLGGGS
LIEYSSRRLMTSLTDVEVAEPLSKLGLEFPDIYLGCYRKSRQGPIIICLTGKDNARIDSAAQALCKKFKK
DVFVEIK*
>A.halleri v1.1|Araha.32690s0001.1.p
MEIDKAIGESDDKRLKTKYNNAIFVIKRALALYSIEEVAFSFNGGKDSTVLLHLLRAGYFLHKKEQTCSN
GGLSSFPVRTIYFESPSAFTEINAFTYDAAQTYNLQLDIIRQDFKSGLEALLKANPIRAIFLGVRIGDPT
AVGQEQFSPSSPGWPPFMRVNPILDWSYRDVWAFLLTCKVKYCSLYDQGYTSIGSIHDTVPNSLLSVNDT
SSKEKFKPAYLLSDGRLERAGRVKKIASLKYDVETESQKHEVLLASVIAVGDEILSGTVEDQLGLSLCKK
LTFVGWSVQQTTVLRNDIDSVSEEVDRQRSTSDMVFIYGGVGPLHSDVTLAGVAKAFGVRLAPDEEFEEY
LRHLISEQCTGDRNEMAQLPEGITELLHHEKLSVPLIKCRNVIVLAATNTEELEKEWECLTELTKLGGGS
LIEYSSRRLMTSLTDVEVAEPLSKLGLEFPDIYLGCYRKSRQGPIIICLTGKDNARIDSAAQALCKKFKK
DVFVEIK*
>C.rubella v1.1|Carub.0006s0224.1.p
MEIDKAIGESDDKRLKTKYNNAIFVIKRALALYSIEEVAFSFNGGKDSTVLLHLLRAGYFLHKKEQSCSN
GGLSSFPVRTIYFESPSAFTEINAFTYDAAQTYNLQLDIIRQDFKSGLEALLKANPIRAIFLGVRIGDPT
AVGQEQFSPSSPGWPPFMRVNPILDWSYRDVWAFLLTCKVKYCSLYDQGYTSIGSIHDTVPNSLLSVNDT
SSKEKFKPAYLLSDGRLERAGRVKKIASLKNDIETESQKHEVLLASVIAVGDEILSGTVEDQLGLSLCKK
LTSVGWSVQQTTVLRNDIDSVSEEVDRQRSTSDLVFIYGGVGPLHSDVTLAGVAKAFGVRLAPDEEFEEY
LRHLISEQCTGDRNEMAQLPEGITELLHHEKLSVPLIKCRNVIVLAATNTEELEKEWECLTELTKLGGGS
SLLEYSSRRLMTSLTDVEVAEPLSKLGLEFPDIYLGCYRKSRQGPIIICLKGKDNARIDLAAQALCKKFK
KDVLVEIK*
>B.stricta v1.2|Bostr.2128s0110.1.p
MEIDKAIGESDDKRLKTKYNNAIFVIKRALALYSIEEVAFSFNGGKDSTVLLHLLRAGYFLHKKEQSCSN
GGLSSFPVRTIYFESPSAFTEINAFTYDAAKTYNLQLDIIRQDFKSGLEALLKANPIRAIFLGVRIGDPT
AVGQEQFSPSSPGWPPFMRVNPILDWSYRDVWAFLLTCKVKYCSLYDQGYTSIGSIHDTVPNSLLSVNDT
SSKEKFKPAYLLSDGRLERAGRVKKIASLKNDVDTESQKHEVLLASVIAVGDEILSGTVEDQLALSLCKK
LTSIGWSVQQTTVLRNDIDSVSEEVDRQRSTSDMVFIHGGVGPLHSDVTLAGVAKAFGVRLAPDEEFEEY
LRHLISEQCTGDRNEMAQLPEGITELLHHEKLSVPLIKCRNVIVLAATNTEELEKEWECLTELTKLGGGS
SLIEYSSRRLMTSLTDVEVAEPLSKLGLEFPDIYLGCYRKSRQGPIIICLKGKDNARIDSATQALCKKFK
KDVLVEIK*
>M.maritima v1.1|Mamar.0004s0245.1.p
MEIDKAIGESDDKRLKTKYNNAIFVIKRALALYSIEEVAFSFNGGKDSTVLLHLLRAGYFLHKKEQSCSN
GGLSNFPVRTIYFESPSAFTEINAFTYDAAQTYNLQLDIIRQDFKSGLEALLKANPIRAIFLGVRIGDPT
AVGQEQFSPSSPGWPPFMRVNPILDWSYRDVWAFLLTCKVKYCSLYDQGYTSIGSIHDTVPNALLSVNDT
SSKDKFKPAYLLSDGRLERAGRVKKTASLKNDINTESQKHEVLLASVIAVGDEILSGTVEDQLGLSLCKK
LTSAGWCVQQTAVLRNDIDSVSEEVDRQRSTSDMVFIYGGVGPLHSDVTLAGVAKAFGVRLAPDEEFEEY
LRHLISEQCTGDRNEMAQLPEGITELLHHEKLSVPLIKCRNVIVLTATNTEELENEWDCLTELTKLGGGS
SLMELYASRRLMTSLTDVEVAEPLSKLGLEFPDIYIGCYRKSRQGPIIICLKGKDNVRIDSAVQALRKKF
KKDVFVEVK*
>D.sophioides v1.1|Desop.0094s0303.1.p
MEIDKAIGESDDKRLKTKYNNAIFVIRRALALYSIEEVAFSFNGGKDSTVLLHLLRAGYFLHKKELSCAN
GGLSNFPVRTIYFESTSAFTEINAFTYDAAKTYNLQLDIIRQDFKSGLEALLKANPIRAIFLGVRIGDPT
AVGQEQFSPSSPGWPPFMRVNPILDWSYRDVWAFLLTCKVKYCSLYDQGYTSIGSIHDTVPNALLSVNDT
SSKEKFKPAYLLSDGRLERAGRVKKSASLKNDVDTESQKHEVLLASVIAVGDEILSGTVEDQLGLSLCKK
LNSVGWSVQQTAVLRNDIDSVSEEVDRQRSTSDMVFIYGGVGPLHSDVTLAGVAKAFGVRLAPDEEFEEY
LRHLISEQCTGDRNEMAQLPEGITELLHHEKLSVPLIKCRNVIVLAATNTEELEKEWECLTELTKLGGGS

SLIELYVSRRLMTSLTDVEVAEPLSKLGLEFPDIYLGCYRKSRQGPIIICLKGKDNARIDSAVQALCKKF
KKDVLVEVK*
>L.sativum v1.1|Lesat.0156s0190.1.p
MEIDKAIGESDDKRLKTKYNNAIYVIRRALALYSIEEVAFSFNGGKDSTVLLHLLRAGYFLHKKEQSCSN
GGLSSFPVRTIYFESPSAFTEINAFTYDAAQTYNLQLDIIRQDFKSGLEALLKANPIRAIFLGVRIGDPT
AVGQEQFSPSSPGWPPFMRVNPILDWSYRDVWAFLLTCNVKYCSLYDQGYTSIGSIHDTVPNALLSVNDT
SNKEKFKPAYLLSDGRLERAGRVKKIASINNDTDTESHKHEVLLASVIAVGDEILSGTVEDQLGLSLCKK
LTSVGWSVQQAAVLRNDIDSVSEEVDRQRSTSDMVFIYGGVGPLHSDVTLAGIAKAFGVRLAPDEEFEEY
LRHLISEQCTGDRNEMAQLPEGITELLHHEKLSVPLIKCRNVIVLAATNAEELEKEWYCLTELTKLVGGS
SLVEIYVSRRLMTSLTDVEVAEPLSKLVLEFPDIYLGCYRKSRQGPIIICLKGKDNARIDSAVQVLRKKF
KKDVFVEIK*
>E.salsugineum v1.0|Thhalv10013303m
MEIDKAIGESDDKRLRTKYNNAIFVIKRALALYSVQLVDFCSIEEVAFSFNGGKDSTVLLHLLRAGYFLH
KKELSCSNGGLSKFPVRTIYFESPSAFTEINAFTYDAAQTYNLQLDIIRQDFKSGLEALLKANPIRAIFL
GVRIGDPTAVGQEQFSPSSPGWPPFMRVNPILDWSYRDVWAFLLTCKVKYCSLYDQGYTSIGSIHDTVPN
ALLSVNNASNKEKFKPAYLLSDGRLERAGRVKKVASIKNDINSDSQKHEVLLASVIAVGDEILSGTVEDQ
LGLSLCKKLTSVGWSVQQTSVLRNDIDSVSEEVDRQRSTSDMVFIYGGVGPLHSDVTLAGVAKAFGVRLA
PDEEFEEYLRHLISEQCTGDRNEMAQLPEGITELLHHEKLSVPLIKCRNVIVLAATNTEELEKEWECLTE
LTKLGGSSSLIELYASRRLMTSLTDVEVAEPLSKLGLEFPDIYLGCYRKSRQGPIIICLKGKDNARVDSA
VQALCKKFKQSVFVEIK*
>B.rapaFPsc v1.3|Brara.J02819.1.p
MEIDKAIGESDDKRLKTKYNNAIFVIKRALSLYSIEEVAFSFNGGKDSTVLLHLLRAGYFLHKKELSCSN
GGLSSFPVRTIYFESPSAFTEINAFTYDAAQTYGIQLDIIRQDFKSGLEALLKANPIRAIFLGVRIGDPT
AVGQEQFSPSSPGWPPFMRVNPILDWSYRDVWAFLLTCKVKYCSLYDQGYTSIGSIHDTVPNALLSVNDT
SSKEKFKPAYLLSDGRLERAGRVKKNASTKNDVGSDSQKHEVLLASVIAVGDEILSGTVEDQLGLSLCKK
LTSVGWSVQQTSVLRNDIDSVSEEVDRQRSICDMVFIYGGVGPLHSDVTLAGVAKAFGVRLAPDEEFEEY
LRHLISEHCTGDRNEMAQLPEGITELLHHEKLSVPLIKCRNVIVLAATNTEELEKEWECLTELTKLGGST
SLMELYASRRLMTSLTDVEVAEPLSKLGLEFPDIYLGCYRKSRQGPIIICLKGKDNARIDSAVEALRKKF
KEGVFVDMK*
>C.maritima v1.1|Camar.0141s0022.1.p
MEIDKAIGESDDMRLKTKYNNAIFVIKRALSLYSIEEVAFSFNGGKDSTVLLHLLRAGYFLHKKEMSCSN
GGLSSFPVRTIYFESPSAFPEINSFTYDAAQTYDLQLDIIRQDFKSGLEALLKANPIRAIFLGVRIGDPT
AVGQEQFSPSSPGWPPFMRVNPILDWSYRDVWAFLLTCKVKYCSLYDQGYTSIGSIHDTVPNALLSVNDT
SSKEKFKPAYLLSDGRLERAGRVKKNASLKNDIDSDSQKHEVLLASVIAVGDEILSGTVEDHLGLYLCKK
LTSVGWSVQQTSVIRNDIDSVSEEVDRQRSICDMVFIYGGVGPLHSDVTLAGVAKAFGVRLAPDEEFEEY
LRHLISEQCTGDRNEMAQLPEGITELLHHEKLAVPLIKCRNVIVLAATNTEELEKEWECLTELTKLGGST
SLMELYASRRLMTSLTDVEVAEPLTKLGLEFPDIYLGCYRKSRQGPIIICLKGKDNARIDSAVQALCKKF
KEGVFVDMK*
>B.oleraceacapitata v1.0|Bol010052
MEIDKAIGESDDKRLKTKYNNAIFVIRRALSLYSIEEVAFSFNGGKDSTVLLHLLRAGYFLHKKELSCSN
GGLSSFPVRTIYFESPSAFTEINAFTYDAAQTYGIQLDIIRQDFKSGLEALLKANPIRAIFLGVRIGDPT
AVGQEQFSPSSPGWPPFMRVNPILDWSYRDVWAFLLTCKVKYCSLYDQGYTSIGSIHDTVPNALLSVNDA
SSKEKFKPAYLLSDGRLERAGRVKKNASTKNDVGSDSQKHEVLLASVIAVGDEILSGTVEDQLGLSLCKK
LTSVGWSVQQTSVLRNDIDSVSEEVDRQRSICDMVFIYGGVGPLHSDVTLAGVAKAFGVRLAPDEEFEEY
LRHLISEHCTGDRNEMAQLPEGITELLHHEKLSVPLIKCRNVIVLAATNTEELEKEWECLTELTKLGGST
SLMELYASRRLMTSLTDVEIAEPLSKLGLEFPDIYLGCYRISRQGPIIICLKGKDNARIDSAVQALCKKF
KEGVFVDMK*
>I.tinctoria v1.1|Isati.0576s0008.1.p
MEIDKAIIESDDKRLKTKYNNAIFVIRRALALYSIEEVAFSFNGGKDSTVLLHLLRAGYFLHKKELSCSN
GGLSNFPVRTIYFESPSAFTEINAFTYDAAQTYDLQLDIIRQDFKSGLEALLKANPIRAIFLGVRIGDPT
AVGQEQFSPSSPGWPPFMRVNPILDWSYRDVWAFLRTCKVKYCSLYDQGYTSIGSIHDTVPNALLSVNDT
SSKEKFKPAYLLSDGRLERAGRVKKNASIKNDVDSDSQKHEVLLASVIAVGDEILSGTVEDQLGLSLCRK

LTSVGWSVQQTAVLRNDIDSVSEEVDRQRSICDMVFIYGGVGPLHSDVTLAGVAKAFGVRLAPDEEFEEY
LRHLISEQCTGDRNEMAQLPEGITELLHHEKLSVPLIKCRNVIVLAATNTEELEKEWECLTELNKLGGST
SLMEPYASRRLMTSLTDVEVAEPLSKLGLEFPDIYLGCYRKSRQGPIIICLKGKNSARIDSAVQALCKKF
KEGVFVDMK*
>M.perfoliatum v1.1|Myper.0034s0398.1.p
MEIDKAIGESDDKRLKTKYNNAIFVIRRALALYSIEEVAFSFNGGKDSTVLLHLLRAGHFLHKKELSCSN
GGLSNFPVRTIYFESPSAFDEINAFTYDAAQTYDLQLDIIRQDFKSGLEALLKANPIRAIFLGVRIGDPT
AVGQEQFSPSSPGWPPFMRVNPILDWSYRDVWAFLRTCKVKYCSLYDQGYTSIGSVHDTVPNALLSVNDT
SGKEKFKPAYLLSDGRLERAGRVKKNASIKNDVDSDSQKHEVLLASVIAVGDEILSGTVEDQLGLSLCKK
LTSVGWSVQQTAVLRNDIDSVSEEVDRQRSICDMVFIYGGVGPLHSDVTLAGVAKAFGVRLAPDEEFEEY
LRHLISEQCTGDRNEMAQLPEGITELLHHEKLSVPLIKCRNVIVLAATNTEELEKEWECLTELPKLGGST
SLMELYASRRLMTSLTDVEVAEPLSKLGLEFPDIYLGCYRKSRQGPIIICLKGKNSARIDSAVQALCKKF
KEGVFVDLK*
>S.alba v1.1|Sialb.0536s0021.1.p
MEIDKAIGESDDKRLRTKYNNAIFVIRRALSLYSIEEVAFSFNGGKDSTVLLHLLRAGYFLHKKELSCSN
GGLSSFPVRTIYFESPSAFTEINAFTYDAAQTYDLQLDIIRQDFKSGLEALLKANPIRAIFLGVRIGDPT
AVGQEQFSPSSPGWPPFMRVNPILDWSYRDVWAFLLTCKVKYCSLYDQGYTSIGSIHDTVPNALLSVNDT
SSKEKFKPAYLLSDGRLERAGRVKINASIKNDIGSDSQKHEVLLASVIAVGDEILSGTVEDQLGLSLCKK
LTSVGWWVQQTSVLRNDIDSVSEEVDRQRSICDMVFIYGGVGPLHSDVTLAGVAKAFGVRLAPDEEIEEY
LRHLISEQCTGDRNEMAQLPEGITELLHHEKLSVPLIKCRNVIVLTATNTEELEKEWECLIQLTKLGGST
SLMELFASRRLMTSLTDVEVAEPLSKLGLEFPDVYLGCYRKSRQGPIIICLKGKDNARIDSAVQALRKKF
KEGVFVDMK*
>E.vesicaria v1.1|Eruve.1836s0006.1.p
MEIDKAIGESDDKRLKTKYNNAIFIITRALSLYSIEEVAFSFNGGKDSTVLLHLLRAGYFLHKKEMGSSN
GGLTSFPVRTIYFESPSAFTEINAFTYDAAQTYGIQLDIIRQDFKSGLEALLKANPIRAIFLGVRIGDPT
AVGQEQFSPSSPGWPPFMRVNPILDWSYRDVWAFLLTCKVKYCSLYDQGYTSIGSIHDTVPNALLSVNDT
SSKEKFKPAYMLSDGRLERAGRVKKNASIKNDVGSDSQKHEVLLASVIAVGDEILSGTVEDQLGLSLCKK
LTSVGWSVQQTSVIRNDIDSVSEEVDRQRSICDMVFIYGGVGPLHSDVTLAGVAKAFGVRLAPDEEFEEY
LRHLISEHCTGDRNEMAQLPEGITELLHHEKLSVPLIKCRNVIVLAATNTDELEKEWECLTELTKLGGST
SLIDASRRLMTSLTDVEVAEPLSKLGLEFPDIYLGCYRKSRQGPIIICLKGKDNARIDSAVQALCKKFKE
GVFVDI*
>C.violacea v1.1|Clevi.0016s0355.1.p
MEIDKAIGECEDKRLKTKYNNAIHVIKRALALYSIEEVAFSFNGGKDSTVLLHLLRAGYFLHKKELSCSN
GGLSLFPIRTIYFESPSAFPEINAFTYDTAQTYNLQLDIIRQDFKSGLEALLEANPIRAIFLGVRIGDPT
AVGQEQFSPSSPGWPPFMRVNPILDWSYRDIWAFLLTCKVQYCSLYDQGYTSIGSIHDTVPNALLRFNDS
NSKENFKPAYLLSDGRLERAGRVKKVSSARANSPSIGNDLDTESHKHKVLSASVIAVGDEILSGTVEDQL
GPSLCKKLSYVGWSVQQTAVLRNDIDSVAEEVDRQRSTNDMVFIYGGVGPLHSDVTLAGVAKAFGVRLAP
DEEFEEYLRHLIGEHCTGDRNEMALLPEGITELLHHEKLSVPLIKCCNVIVLTATNTCELDREWECLTEL
TKLGGVLAHMEPYASRHLMTSLTDVEVAEPLSKLCLEFPDIYLGCYRKSRQGPLVVCFKGKDKARIDSAA
EALRKKFKEGVFVEIE*
>S.purpurea v5.1|Sapur.006G101200.1.p
MEIDKAIGESDDRRLKTKYNNAIYVIKRALALYSIEEVAFSFNGGKDSTVLLHLLRAGYFLHKMEQKCSN
GGLTSFPIRTIYFENSSAFPEINSFTYDTASSYGLQLDIISSDFKSGLEKLLKANPIRAIFLGVRIGDPT
AVGQEQFSPSSPGWPPFMRVNPILDWSYRDVWAFILTCKVQYCSLYDQGYTSIGSIHDTVPNALLSISDS
CCKEKFKPAYLLSDGRLERAGRAKKFSPSITAVGNGSGGVESHKSSLLVASAIAVGEEILFGTVEDQLGL
SLCRKIHSIGWSVSQTIVVRNDKDSVAEEVERRKSTNDMVFIYGGVGPLHSDVTLAGVAKAFGVRLAPDE
EFEEYLRHLISDHCTGDRNEMALLPEGITELLHHEKLAVPLVKCQNVIIFTATNATELDEEWDCLIELTR
SSGLLSTMVPYVSKHLQTNLSDVETAQPLSKLCLEFPDLNIGCYRKSRKGPLLISFEGKDQTQIDSAVES
LCKKFHPGTFSEIH*
>T.cacao v2.1|Thecc.05G068900.1.p
MEIDKAIRESDDTRLKTKYNNAVYVIKRALALYSIEEVAFSFNGGKDSTVLLHLLRAGYFLHRREQNCPN
GVLTDFPIRTIYFESASAFPEINSFTYDIAKMYGMQMDIIRSDFKSGLEMLLKAKPIRAIFLGVRIGDPT

AVGQEQFSPSSPGWPSFMRVNPILDWSYRDVWAFLLTCKVQYCSLYDQGYTSIGSIYDTVPNALLSISNT
SSKEKFKPAYLLPDGRLERAGRVKKISSSDGTRLPAINNGLDSVDSHRNRMFTASVIGVGDEILFGTVED
QLGRSLCRKLHSISWLTSQTAVVRNDIDSVAEEIERRKSTNDVVFLYGGVGPLHSDVTLAGVAKAFGVRL
APDEEFEEYLRHLIGDHCTGDRNEMAQLPEGITELLHHEKLPVPLIKCRNVIVLSATNATELDKQWDCLI
ELTGSDGCLVTMEPYSSKRLTTNLTDVETAQPLSKLCLEFPDLYIGCFRESRQGPLVISFEGKDLARIHA
AVETLCKKLHPEAFSEVN*
>C.clementina v1.0|Ciclev10011527m
MEIDKAIRESDDRRLKTKYNNAINVIQRTLALYSIEEVAFSFNGGKDSTVLLHLLRAGYFLHKGEQSCSN
GSLTFPIRTIYFESNSAFPEINSFTYDTASKYVLQLDIIRSDFKSGLEALLNAKPIRAIFLGVRIGDPTA
VGQEQFSPSSPGWPPFMRVNPILDWSYRDVWAFILTCKVQYCSLYDQGYTSIGSIHDTVPNALLCVSDSS
NNQEKFKPAYMLSDGRLERAGRVKKVSPSICGTAVANVMDNVDSHKSSLLKASAIAVGDEILFGTIEDQL
GPSLCKKLHSIGWSVSQIAVLQNDIDSVAEEVERQKASHDMVFVYGGVGPLHSDVTLAGVAKAFGVRLAP
DEEFEEYLRQLIGDRCTGDRNEMALLPEGTTELLHHDKLLLPLIKCQNVIILTATNVTELDKEWNCLIEL
LRSGGLSLMEPYTSKSLTTNLSDLEAAQPLSKLCLEFPDLHIGCYRKSRQGPLIISFEGKDQARIEAAIE
SLFKKFHRGAFSEVV*
>G.raimondii v2.1|Gorai.009G414300.1
MEIDKAITESDDKRLKTKYNNAIYVIKRALALYSIEEVAFSFNGGKDSTVLLHLLRAGYFLHRVEQSCSN
GVQIDFPIRTIYFESTSVFPEINTFTYDTSKTYGLQMDIIRTDFKSGLEALLKSKPIRAIFLGVRIGDPT
AVGQEQFSPSSPGWPPFMRVNPILDWSYRDVWAFLLTCKVQYCSLYDQGYTSIGSIYDTVPNALLSISST
SSKEKFKPAYLLPDGRLERAGRVKKNSPLDGTRSPAVNNGLDSVNSHKNRMFTASVIGVGDEILFGTVED
QLGPLLCKKLHSIGWMTSRSTVVRNDIDSVAEEVDRCKSVSDLVFLYGGVGPLHSDVTSAGVAKAFGVRL
APDEEFEEFLRHLIGDHCTGDRNEMAQLPEGITELLHHEKLPVPLIKCCNVIVLSATNATELEKQWDCLI
ELTESDGFLVTIESYSSKRLTTNLTDVETAQPLSKLCLEFPDLYIGCFRRSRQGPLVISFEGKDPSRVQA
GVEALCKKFNAGAFSEVN*
>C.dentata v1.1|Caden.09G036900.1.p
MEIDKAIRESNDRRLKTKYNNAIYVIKRALALYSIEEVAFSFNGGKDSTVLLHVLRAGYFLHKAEQSCSN
EGLNDFPIRTIYFESTSAFPEINSFTYDAASAYGLQMDIIRTDFKSGLEDLLKTKPIRAIFLGVRIGDPT
AVGQEQFSPSSPGWPSFMRVNPILDWSYRDVWAFILTCKVQYCSLYNQGYTSIGSIYDTVPNALLCISDS
SNSKETFRPAYLLSDGRLERAGRVKKFSPVSNGPDNIDLRKNSMLTASVIAVGDEILFGTIEDQLGPSLC
RKLHSIGWSVSQATVVRNDVDSVAEEVERQKSTNDLIFIYGGIGPLHSDVTLSGVAKAFGVRLAPDEEFE
EYLRHLIGDQCTGDRNEMAQLPEGITELLHHENLPVPLMKCQNVIILTATNIMELDKEWECLIELTSSGG
LFEMMEPYISKCLITQLSDVETAQPLSKLCLEFPDLYIGSYRRSRTGPLTISFRGKDQARIESAMEALYK
KFHPGVFSETN*
>G.hirsutum v1.1|Gohir.D04G110200.1.p
MEIDKAIRESDDKRLKTKYNNAIYVIKRALALYSIEEVAFSFNGGKDSTVLLHLLRAGYFLHRVEQSCSN
GVQIDFPIRTIYFESTSVFPEINTFTYDTSKTYGLQMDIIRTDFKSGLEALLKSKPIRAIFLGVRIGDPT
AVGQEQFSPSSPGWPPFMRVNPILDWSYRDVWAFLLTCTVQYCSLYDQGYTSIGSIYDTVPNALLSISST
SSKEKFKPAYLLPDGRLERAGRVKKNSPLDGTRSPAVNNGLDSVNSHKNRMFTASVIGVGDEILFGTVED
QLGPLLCKKLHSIGWMTSRSTVVRNDIDSVAEEVDRCKSVSDLVFLYGGVGPLHSDVTSASVAKAFGVRL
APDEEFEEFLRHLIGDHCTGDRNEMAQLPEGITELLHHEKLPVPLIKCCNVIVLSATNATELEKQWDCLI
ELTGSDGFLVTIESYSSKRLTTNLTDVETAQPLSKLCLEFPDLYIGCFRRSRQGPLVISFEGKDPSRVQA
GVEALCKKFNAGAFSEVN*
>R.communis v0.1|30078.m002280
MSEYMEIDKAISESDDRRLKTKYNNAIYVIQRALALYSVEEVAFSFNGGKDSTVLLHLLRAGYFLYKGEK
SCSNGGLTSFPIRTIYFESSSAFPEINSFTHDTASSYGLQLDIISSDFKSGLENLLKANPIRAIFLGVRI
GDPTAVGQEQFSPSSPGWPPFMRVNPILDWSYRDVWAFILTCKVQYCSLYDQGYTSIGSIHDTIPNALLS
IRDSSCKEKFKPAYMLADGRLERAGRVKKLSPPTVGHLPGVSDSPDNVDAHKNSALVAAVVAVGDEILFG
AVEDQLGPSLCRKLHCIGWSVSQTAVVRNDVDSVAAEVEQRKSTNDMVFIYGGVGPLHSDVTLAGVAKAF
GVRLAPDEEFEEYLRHLIGDHCTGDRNEMALLPEGITELLHHDKLPVPMIKCQNVIILAATNIGELDREW
ECLTEFTRSYGLLATMEPFAAKRLTTNISDVEIAQPLSKLCLEFPDLNIGVYRKSRNGTLIITFEGKKQA
RIDSAVEALRKKFHPGVFSEMN*
>P.trifoliata v1.3.1|Ptrif.0006s0330.1.p

MEIDKAIRESDDRRLKTKYNNAINVIQRTLALYSIEEVAFSFNGGKDSTVLLHLLRAGYFLHKGEQSCSN
GSLTFPIRTIYFESNSAFPEINSFTYDTASKYVLQLDIIRSDFKSGLEALLNAKPIRAIFLGVRIGDPTA
VGQEQFSPSSPGWPPFMRVNPILDWSYRDVWAFILTCKVQYCSLYDQGYTSIGSIHDTVPNALLCVSDSS
NNQEKFKPAYMLSDGRLERAGRVKKVSPSICGTAVANVMDNVDSHKSSSLKASAISVGDEILFGTIEDQL
GPSLCKKLHSIGWSVSQIAVLQNDIDSVAEEVERQKASHDMVFVYGGVGPLHSDVTLAGVAKAFGVRLAP
DEEFEEYLRQLIGDHCTGDRNEPYNLGSQETFGIQISQMALLPEGTTELLHHDKLLLPLIKCQNVVILTA
TNVTELDKEWNCLIELLRSGGLSLMEPYTSKSLTTNLSDLEAAQPLSKLCLEFPDLHIGCYRKSRQGPLI
ISFEGKDQARIEAAIESLFKKFHRGAFSEVV*
>P.persica v2.1|Prupe.7G128300.1.p
MEIDKAITECDDRRLKTKYSNAIYVIQRTLALYSIEEVAFSFNGGKDSTVLLHLLRAGYFLHKGEKSCSN
GDVKDFPIRTIYFESPSAFPEINSFTYDTATTYGLQLDIIRSDFKSGLEALLNAKPIKAIFLGVRMGDPT
AVGQEQFSPSSLGWPPFMRVNPILDWSYRDVWAFLLTCKVPYCSLYDQGYTSIGSIYDTVPNALLCINNS
SGSKEVFRPAYLLSDGRLERAGRVKKLPSSVSGHNPAVINGLDNMDLHNRSLLTASAIAVGDEILFGTVE
DQIGPSLCRKLHSFGWLVSQTAVVRNDIDSVAEEVERRQSTDDMVFIYGGVGPLHSDVTTAGVAKAFGVR
LAPDEEFEEYLRHLIGDQCTGDRNEMALLPEGITELLHHEKLIVPLIKCKNVIILTATNALELDEEWNCL
IELMTSDDVLLMMQSFVSKHLTTNLTDVEIARPLSKLCLEFPDLYIGCHRKSRKEPLVLYFKGKDQDRIE
SAVEALNKKFCPGAFVEINSS*
>A.occidentale v0.9|Anaoc.0001s0598.1.p
MEIDKAIRESDDLRLKTKYNNAIYVIRRALALYTIEEVAFSFNGGKDSTVLLHLLRAGWFLHKGEQSCSN
GNLNNFPIGTIYFESSCAFPEINSFTYDTAKLYGLQLDIIRTDFKSGLEALLKVKPIRAIFLGVRIGDPT
AVGQEQFSPSSPGWPAFMRVNPILDWSYRDVWAFILTCKVPYCSLYDQGYTSIGSIYDTVPNALLCVNNS
ANNKEKFKPAYLLSDGRCERAGRAKKLSQPVCGHHPAIANGMENVDSHRSSVLTASVIAVGDEILFGTVE
DQLGPLLCKKLHSIGWKVRQTAVVQNDVDSVAEEVDRQKSANDMVFMYGGVGPLHSDVTSAGVAKAFGVR
LAPDEEFEEYLRHLIGKHCTGDRNEMALLPEGITELLHHEELPVPMIKCQNVIILSSSNVTELDKEWKCL
IEKLTSSGLSLMEPYTSKSLSTNLSDVELAQPLSKLCLEFPDLYIACFRKSRQGSLIIRFEGKDEARIES
AVESLGKKFHPGTFSEVA*
>P.trichocarpa v3.1|Potri.006G123000.1.p
MEIDKAIRESDDRRLKTKYNNAIYVIKRALALYSIEEVAFSFNGGKDSTVLLHLLRAGYFLHKMEQKCSN
GGLTSFPIRTIYFESSAAFPEINSFTYDTASSYGLQLDIISSDFKSGLEKLLKANPIRAIFLGVRIGDPT
AVGQEQFSPSSPGWPPFMRVNPILDWSYRDVWAFILTCKVQYCSLYDQGYTSIGSIHDTVPNALLSISDS
CCKEKFKPAYLLSDGRLERAGRAKKFSSSINAVGNGSGSVDSHKSSLLVASVIAVGEEILFGTVEDRSVS
QTVVVQNDKDSVAEEVERRKSTNDMVFIYGGVGPLHSDVTLAGVAKAFGVRLAPDEEFEEYLRHLISDHC
TGDQNEMALLPEGITELLHHEKLAVPLIKCQNVIIFTATNATELDKEWDCLIELTRSCGFLPTMVPYVSK
HLQTNLSDVETAQPLSKLCLEFPDLNIGCYRKSRKGPLIISFEGKDQTQIESAVESLCKLFHPGTFSGIH
*
>M.esculenta v7.1|Manes.12G084800.3.p
MEIDKAIRECDDRRLKTKYNNAIYVIQRALTLYSIEEVAFSFNGGKDSTVLLHLLRAGYFLHKEAQNCSD
RGLNCFPIRTIYFESNSAFPEINSFTYDTASRYGLQLDIISLDFKCGLESLLKANPIKAIFLGVRIGDPT
AVGQEQFSPSSPGWPPFMRVNPILDWSYRDVWAFILTCKVQYCSLYDQGYTSIGSIYDTIPNSMLCINDS
GCNTKFKPAYLLSDGRLERAGRVKKSSPSIPGPFSVAGDISDSVDPHKNGMLVASLIAVGDEILSGTVED
QLSTSLCRKLLSIGWSVSQTVIVQNDIDSVAEEVERHKSTNDMVFIYGGVGPLHSDVTSAGVAKAFGVRL
APDEEFEEYLKHLIGDYCTGDRNEMALLPEGITELLHHEKLLVPMIKCQNVIILAATDSAELDKEWECLT
ELGKSYGLLAIGEPFISRRLTTNLSDVEIAQPLSKLCLEFPDVSIGAYRKCRRNGSLIINFKGKKRARID
LAVEALCKMFPSIAFTEVN*
>L.albus v1|Lalb_Chr25g0288951
MEIDKAIRECDDQRLQTKYNNATYVIQRALALYSIEEVAFSFNGGKDSTVLLHLLRAGYFLHKRGQSNAN
GDLKDFSMRTIYFESPCAFPEINSFTYDIAATYDLQIDTIRLDFKSGLEALLKENPIRAIFLGVRIGDPT
AVGQEQFSPSSPGWPPFMRVNPILDWSYRDVWAFLLTCKVKYCSLYDEGYTSIGSIYDTVPNSLLSISNS
PDKFKPAYLLADGRLERAGRAKKLSSTSGQLHVDSNGLTDLDLHKNSMLTASIIVVGDEILFGTVEDQLG
PYLCRKLHSVGWSVLQLSVVHNNIDSVAEEVEQRKSTCDTVFIYGGVGPLHSDVTLAGIAKAFGVRLAPD
EEFEEYLRHIIGDQCTGDRNEMAQLPEGITELLHHDKLSMPLIKCHNIIVLNAANVSELENQWDCLIELT

KSCDMLTLLEPYVSKQVTTKLSDVEIAQPLSKLCLEYPDLYIGCYRKARYGSVIVSFKGKDEARLETAIK
ALHNKFQPGAFIEMK*
>F.vesca v4.0.a2|FvH4_6g16530.t5
MEIDKAIKECDDRRLKTKYNNAIYVIQRALALYSIEEVAFSFNGGKDSTVLLHLLRAGYFLHKGEQSCTN
GGVQDFPIRTIYFDCPSAFPEINSFTYDAANTYGLQLDIIRSDFKSGLEALLKSKPIRAIFLGVRMGDPT
AVGQEQFSPSSLGWPPFMRVNPILDWSYRDVWAFLLTCKVHYCSLYDQGYTSIGSIYDTVPNSLLSINNS
SSSKEAFRPAYLLSDGRLERAGRVKKLPHSVGGKKPVVANGLTSMDLPKSSLLMASAIAVGDEILFGTAE
DQLGHSLCKKLHSIGWSVSQTTVVRNEVDSVAEEVERRQSTNDMVFIYGGVGPLHSDVTLAGVAKAFGVR
LAPDEEFEEYLRHLIGDQCTGDRNEMALLPEGITELLHHEKLMVPLIKCKNVIIFTATNVLELDDEWNCL
IELMRSGGELVMAQPFVSKCLTTNLADLEVAQPVSKLCLEFPDLYIGCYRISRREPLRIYFEGKDQNRIE
SAKEALCKKFQPGAFSEINLS*
>P.acutifolius_WLD v2.0|Phacu.WLD.001G214100.1
MEIDRAIRECDDRRLQTKYKNATYVVQRALTLYSIEEVAFSFNGGKDSTVLLHILRAGYFLHKKGQNSVN
GDLKDFPIRTIYFESPCAFPEINSFTYDTAATYGLQIDTISLDFKSGLEALLKEKPIRAIFLGVRIGDPT
AVGQEQFSPSSPGWPPFMRVNPILDWSYRDVWAFLLTCKVNYCSLYDQGYTSIGSIHDTVPNSLLCVSNS
SYKFKPAYLLADGRLERAGRAKRSSSTGGQLPVDSNGLTSLDSHKNSMLTASIIAVGDEILFGIVEDQLG
PYLCRKLHSIGWSVFKLSVVHNNIDSVAEEVERQKSKSDMVFIYGGVGPLHSDVTIAGIAKAFGVRLAPD
EEFEEYLRHIIGDQCTGDRNEMAQLPEGITELWHHDKLSVPLIKCQNVIILSATNVSELEKQWDCWIELA
KSSDLLALLEPYVSKHVATNLTDVEIAQPLSKLCLEFPDLYIGCYRKARYGSVIVSLKGKDLTRIDSAIK
ALHKKFQPGAFIEMN*
>P.vulgaris v2.1|Phvul.001G179700.1.p
MEIDRAIRECDDRRLQTKYKNATYVVQRALTLYSIEEVAFSFNGGKDSTVLLHILRAGYFLHKKGQNSVN
GDLKDFPIRTIYFESPCAFPEINSFTYDTAATYGLQIDTISLDFKSGLEALLKEKPIRAIFLGVRIGDPT
AVGQEQFSPSSPGWPPFMRVNPILDWSYRDVWAFLLTCKVNYCSLYDQGYTSIGSIHDTVPNSLLCVSNS
SYKFKPAYLLADGRLERAGRAKRPSTGGQLPVDSNGLTSLDSHKNSMLTASIIVVGDEILFGIVEDQLGP
YLCRKLHSIGWSVFKLSVVHNNIDSVAEEVERQKSKSDMVFIYGGVGPLHSDVTIAGIAKAFGVRLAPDE
EFEEYLRHIMGDQCTGDRNEMAQLPEGITELWHHDKLSVPLIKCQNVIILSATNVSELEKQWDCWIELAK
SSDLLALLEPYVSKHVATNLTDVEIAQPLSKLCLEFPDLYIGCYRKARYGSVIVSFKGKDLTRIDSAIKA
LHKKFQPGAFIEMN*
>D.carota v2.0|DCAR_015004
MEIDKAVRESDDQRLKNKYNNAIYVIRRALALYSIEEVAFSFNGGKDSTVLLHLLRAGYYLHKEEKSCAS
VGLVDHFEFPIRTIYFETPSTFTEINSFTYETASTYGLQMDIIRLDFKSGLEALLKANPIKAVFLGVRIG
DPTAVGQEQFSPSSPGWPSFMRVNPILDWSYRSSIIFDRDVWAFLLTCKVQYCSLYDQGYTSIGSIHDTV
PNDLLCIQDSKDDKRSFKPAYLLPDGRLERAGRAKKFSANPMKSPVISNGDIKTVDIHHNSLHTASVIAV
GDEILSGIVEDKVGHSLCRKLCSIGWAVARMSVVRNDIDSVAEEVERCKGKSDMVFVYGGVGPLHSDVTV
SGVAKAFGVRTAPDEEFEEYLRHLIGEKCTGDRNEMAQLPEGITELLHHEKLPTPLIKCLNVIILAATNI
TELDVQWDCLTELTRSSGLLSSIEPFVSKRLAMNISDVKAAQPLSKLPLEFPDIYIRCYRESRNGPLIVC
LEGKDQARIEVAAEALSKKFEPGVLDINQQS*
>L.japonicus Lj1.0v1|Lj1g0000599.1
MEIDRAIRECDDRRLQTKYNNATYVIQRALALYSIEEVAFSFNGGKDSTVLLHLLRAGYFLHKEGQNSAN
GDLKDFPIRTIYFESPCAFPEINSFTYDTAATYGLQIDTIRLDFKSGLEALLKEMPIRAIFLGVRIGDPT
AVGQEQFSPSSPGWPPFMRVNPILDWSYRDVWAFLLICKVNYCRLYDQGYTSIGSIYDTVPNSLLCISNS
SNKFKPAYLLADGRLERAGRVKKLSSSTCGQLPVDSNGLTSLDMHKNSMLTASIIGVGDEILFGIVEDQL
GPYVCRKLHSVGWSVLQLSVAHNNIDSVAEEVERQKSTTDMVFIYGGVGPLHSDVTLAGIAKAFGVRLAP
DEEFEEYLRQTIGDQSTGDRNEMAQIPEGITELLHHEELSVPLIKCQNVIVLSATNVLELEKQWDCLMEL
TKSSNLLKLLDPYVSKNVTTNLLDTEVAEPLSKLCLEFPDLYIGCYRKARYGSLIVSFRGKDQTRIESAI
KALQKKFQPGAFMEMN*
>G.soja v1.1|GlysoPI483463.03G146700.1.p
MEIDKAIRECDDRRLQTKYNNATYVVQRALALYSIEEVAFSFNGGKDSTVLLHILRAGYFLHKKGQNSVN
GDLKDFPIRTIYFESPCAFPEINSFTYDTAAIYGLQIDTISLDFKSGLEALLKEKPIRAIFLGVRIGDPT
AVGQEQFSPSSPGWPPFMRLNPILDWSYRDVWAFLLTCKVNYCSLYDQGYTSIGSIYDTVPNSLLCISNS
SNKFKPAYLLADGRLERAGRAKRPSTSTGGQIPAESNGLTSQDSYKNSMLTASIIAVGDEILFGNLEDLL

GPYLCRKLHSIGWSVLQHSAVHNNIDSVAEEVERQKSKSDMVFIYGGVGPLHSDVTIAGIAKAFGVRLAP
DEEFEEYLRHIIGDQCTGDRNEMAQLPEGITELWHHDKLSVPLIKCENVIILSATNVPEMEKQWDCWIEL
AKSSDLLALLEPYVSKHVTTNLSDVEIAQPLSKLCLEFPDLYIGCYRNARYGSLIVSFKGKDLTRIESAI
KALQKKFQPSAFIETK*
>C.sativus v1.0|Cucsa.306680.1
MEIDKAIRDCDDRRLKTKYNNAIYVVKRALALYSTEEVAFSFNGGKDSTVLLHILRAAFFLHKEEEGCSV
DGLKEFPIRTIYFESPSAFPEINSFTYDMATNYGLLMDIIRTDFKSGLESLLKSRPIRAIFLGVRIGDPT
AVGQEQFSPSSPGWPPFMRVNPILDWSYRDVWAFLLTCKVQYCSLYDHGYTSIGSIHDTLPNALLCISNS
TGNEEKFRPAYLLSDGRTERAGRAKRFSPSVLNSMSNGTNNVDLQKQSMLTASVIAVGDEILFGTVEDRL
GLSMRRKVHSIGWSISHTSIVRNDIDSVAEEVELRRSSNEMVFIYGGVGPLFSDATLGGIAKAFGVRLAP
DEEFEEYLRHLIGEHCTGDRNEMAQLPEGITELLHHEKLPVPLIKCHNVIVLTATNLTELDLQWDCLIEL
TRTGDLFPLLEPYKSKHLTTKLSDVEIAPSLAKLCLEFPDIHIGCYREARSGPIIISFKGKNEERNQLAA
EALSKKFQPGAFTDTNLNSPET*
>V.unguiculata v1.1|Vigun01g162500.1.p
MEIDRAIRECDDRRLQTKYKNATYVVQRALTLYSIEEVAFSFNGGKDSTVLLHILRAGYFLHKKGQNSVN
GDLKDFPIRTIYFESPCAFPEINSFTYDTAATYGLQIDTISLDFKSGLEALLKEKPIRAIFLGVRIGDPT
AVGQEQFSPSSPGWPPFMRVNPILDWSYRDVWAFLLTCKVNYCSLYDQGYTSIGSIHDTVPNSLLSVSNS
SYKFKPAYLLADGRLERAGRAKRPSSTGGQLPVDSNGLTSLDSHKNSMLTASIIAVGDEILFGIVEDQLG
PYLCRKLHSIGWSVFKLSVVHNNIDSVAEEVERQKSKSDMVFIYGGVGPLHSDVTIAGIAKAFGVRLAPD
EEFEEYLRHIIGDQCTGDRNEMAQLPEGITELWHHDKLSVPLIKCQNVIILSATNVSELEKQWDCWIELA
KSSDLLALLEPYVSKNAATNLSDVEIAQPLSKLCLEFPDLYIGCYRKARYGSLIVSFKGKDLARIDSAIK
ALHKKFQPGAFIEMN*
>C.arietinum v1.0|Ca_00706
MEIDEAIRGCEDRRLQTKYNNATYVIQRALALYSIEEVAFSFNGGKDSTVLLHLLRAGYFLHKAGQNSAN
GDVKNFPIRTIYFESPCAFPEINSFTYDIAATYGLQIDTISLDFKSGLETLLKEKPIRAIFLGVRIGDPT
AVGQEQFSPSSPGWPPFMRVNPILDWSYRDVWAFLLTCKVNYCSLYDQGYTSIGSIYDTVPNSLLSVSNS
SNKFKPAYLLSDGRLERAGRVKRKSSSAGGQLPNGMDLHKNSTLTASIIAVGDEILFGIVEDQVGPYLCT
KLQSIGWSVLQYSVVHNNIDSVAEEVERQKSRTDMVFIYGGVGPLHSDVTLAGIAKAFDVRLAPDEEFEE
YLRHIIGDQCTGDRNEMAQLPEGITELLHHGKLTLPLIKCQNVIILSATNVSELEKQWDCLIELTKSNDL
LTLLDPYISKHMTTNLSDVEVAQPLSKLCLEFPDICIGCYRKARYGSLIISFKGKDQARIESAIKALQKK
FKSEAFIEMK*
>O.europaea v1.0|Oeu041487.1
MEIDKAIRESDDRRLKTKYKNAIHVIQRALALYSFEEVAFSFNGGKDSTVLLHLLRAGYYLYTSGKYHSI
GDPDDCEITFPIRIIYFESPSAFPEINSFTYDTATAFKLQMDIIRLDFKSGLEALLKAKPIRAIFLGVRI
GDPTAVGQEQFSPSSPGWPPFMRVNPILDWSYRDIWAFLLTCKVRYCSLYDQGYTSIGSIHDTVPNALLS
IGNYNNSEIKFKPAYLLPDGRLERAGRIKKLSSLPLSAISNGLKSEDPHRKCMLTATVIAVGDEILFGTI
EDQLGSTLCRKLHSIGWAVSQFAVTQNDVDSVAEEVERQMSRNDMVFIYGGVGPLHSDVTVAGVAKAFGV
RMAPDEEFEEYLRHLIGEKCTGDRNEMAQLPAGITELLHHEQLRVPLIKCQNVIILTATNVDEMDKEWDC
LIEFMRSSSPLVAVEPFVSKRLATTLSDVEAAHPLSEICIEFPDLYIGGYRESRKGLLVVTFEGKEQERI
NGAVEALRKKFQPGSFSEIN*
>C.arabica v0.5|evm.model.Scaffold_558.31
MEIDIAVRESDDRRLKTKYKNAIYVIQRAFALYSVDEVAFSFNGGKDSTVLLHLLRAGYYLHKAEKIGCN
GDLMDGEIAYPIRTIYFESASAFPEINSFTYETAKSYGLQMEIIRLDFKAGLEALLKAKPIRAIFLGVRI
GDPTAVGQEQFSPSSPGWPPFMRVNPILDWSYRDVWSFLLTCKVRYCSLYDEGYTSIGSVHDTVPNGLLC
IRDSSNSEGKFRPAYLLADGRLERAGRVKKNSSPPCGQLASVSNGLKSRDLSWHSMLTASIIAVGDEILF
GTVEDKLGSSLCRRLHSIGWTVSQLAVTRNDIDSVADEVVKRKSTNDMVFIYGGIGPLHSDVTLAGVAKA
FGVRTAPDEEFEEYLRHLIGEKCTGDRNEMAQLPEGITELLHHEQLPVPLIKCHNVIILSATNVAELDLQ
WDCLLDLSSSNGLLVLMEPLQSKRLCTNTSDVEAAQPLSKLCLEFPDLYIGAYRASRNGPLIITFQGKDQ
GRIAAATAALSEKLHTGQFCESRPRAPSFSPVSVPPSPPPPSPPIPLCSNRYLTKFILSVENAFAEVLTQ
IYRIVSRKAVEASDEGAPSSVPPKGETINIKDEGSSWKR*
>C.quinoa v1.0|AUR62026916-RA

MEIDKAIGDCNDQRLKTKYNNAVYVIQRALALYNIEEVAFSFNGGKDSIVLLHLLRAGYYLHKLEQVKHD
EDLGCNQSKFPIRTIYFESPCAFPEINSFTYETAAIYNLQLDILRQDFKSGLEGLLRTKPIRAIFLGVRI
GDPTAVGQEQFSPSSPGWPPFMRVNPILDWSYRDVWAFLLTSKVPYCSLYDQGYTSIGSVYDTVPNSLLS
VSNPTSGKVEYRPAYMLADGRSERAGRTRKSKVADSSISLSNGTTDVESQQTCIPTASVIGVGDEILLGK
VEDLVGPSLCKKLHSIGWTVTQFDVLRSDIDSVAESVERQKSTSDMVFIYGGIGPLHSDATLSGIAKSFG
VRLAPDEEFEEYLTSIAGYQSTGDKNEMAQLPEGITELLHHEKLPVPLMKCQNVIILMATNLAELDKEWD
CLIELAASSGLLMLTKPFSSKYLSTTLSDVEVAQPLSAMRIDFPDLFIGCYRKSRRGPLVITLEGKDQGK
VQLATEALVKKFPAGTISETI*
>H.annuus r1.2|HanXRQChr14g0454821
MEIDKAIRDSDDRRLKTKYHNAIYVIQRALALYSIEEVAFSFNGGKDSTVLLHLLRAGYFLHQVERGHSN
GGLSDGEFTFPIRTIYFETPSVFPEINSFTYETAASYGVKLDIIREDFKSGLEALLKAKSTKAIFLGVRI
GDPTAVGQEQFSPSSPGWPPFMRVNPILDWSYRDVWAFLLTCKVPYCSLYDQGYTSIGSIHDTVPNALLS
IKDPGVEKQKFRPAYMLSDGRSERAGRARKLSAAAACVPAASNGLKCAEPHQGSIYTASIVAVGDEILFG
IVEDQLGPSLCKKLHSIGWLVSQMATVRCEVDSVAEEVERRKSMSDLVFIYGGVGPLHTDVTVGGVAKAF
GVRLAPDEEYEEYLSHLFGDKCTGNRNEMAQLPEGITELLEHEKLPVPLIKCHNVIVLTATNVDELDRQW
DALIDLRSTNLLAPTGPFVSKHLETSLSDLEVAQPLSKIAIEFPDIYTGCYRKTRAGPLIITLEGKDQET
VEAAMEALSKKLPSGVKQMSV*
>M.guttatus v2.0|Migut.L01711.1.p
MEIDKAIRECDDIRLKTKYNNAIYVIQRALALYSVEEVAFSFNGGKDSTVLLHLLRAGYHLHKSGKNLST
EDAELTFPIRTIYFETKSTFHEINSFTYETASIYKLWMDISRLDFKSGLETLLKANPIRAIFLGVRIGDP
TAVGQEQFSPSSPGWPPFMRVNPILDWSYRDIWAFLLTCKVQYCSLYDQGYTSIGSIHDTVPNALLCDSE
EKYKPAYMLSDGRLERAGRVKKLTSQPLSAVSNGLNHDYSHSKSMFTASVIAVGDEILSGTIEDQIGFVL
CRKLHSISWSVSHIAVARNDIDSIADEVERRKSTNDMVFIYGGVGPLPSDVTVAGVSKAFGVRMAPDEEF
EEYLRHLIGERCTGDRNEMAQLPEGITELVHHEKLSVPLIKCKNVIILTAASVAELENEWDCFIDLMRSN
GVLLTTEPLISKKLATTLPDVEIAQPLSEICRQFPDLYIGTYRKSRGGSIIITFEGNDKARIGAAAEALC
KKFHPEALSETE*
>S.lycopersicum ITAG3.2|Solyc09g008310.3.1
MEIDKAIRECDDRRLKTKYNNAIYVIRRALALYPIQEVALSFNGGKDSTVLLHLLRAGCFLHQAEEFNSG
GDAVDGGKTFPIRTIYFESPSAFPEINSFTYEAASIYDIQMDIIRLDFKSGLEALLKANPIRAIFLGVRI
GDPTAVGQEQFSPSSPGWPPFMRVNPILDWSYRDVWAFLLVSKVRYCSLYDQGYTSIGSIHDTVPNALLC
TRNSDSSEEKFKPAYLLADGRLERAGRAKKNSSAASGKSSSISNGLKMDNLNSGSMLSASVITVGDEILF
GTVEDKLGSVLCKKLHSIGWAVSRVTVTQSDIDSVAEEVERRKSIDDMVFICGGIGPLHSDVTVAGVAKA
FGVRMAPDEEFEEHLRHLIGEKCSGDKNEMALLPEGITELLHHEHLPVPLIKCHNVIILTATNAVELDRQ
WDCLIELAKSNGILELMDPFASKCFATTLSDVEVAQPLSKLCAQFPDLYIGGYRESREGPLIITFEGKDL
SRIEAASQSLCQKFHPGSFSKIE*
>M.domestica v1.1|MD04G1091700
MLFRELLRSTRNIEEVAFSFNGGKDSTVLLHLLRAGYFLHKGEQSCSNECVKDFPMRTIYFESPSAFPEI
NSFTYDAATTYGLQLDIIRSDFKSGLEDLLKSKPIKAIFLGVRIGDPTAVGQDQFSPSSVGWPPFMRVNP
ILDWSYRDVWAFLLTCKVQYCSLYDQGYTSIGSIHDTVPNALLSINNSSDNKEVFRPAYLLSDGRLERAG
RVKKLPPSVRGNKPAAINGLDGVGLHKSGLLMASAIAVGDEILFGTVEDQLGPSLCRKLHSIGWSVSQTV
VVRNDVDAVAEEVEQRQSTDDMVFMYGGVGPLHSDVTLAGVAKAFAVRLAPDEEFEEYLRHLIGDQCTGD
RNEMALLPEGITELLHHEKLIVPLIKCKNVIIFTATNVLELDEQWNCLIELMISDGGLATMQPFVSRHLT
TNLTDVEIAQPLSKLCLEFPDLYIGCRRKSRKEPLIIYFEGKDQDQIQSAIEALCKKFHPGAFVETAVVQ
LVF*
>V.vinifera v2.1|VIT_208s0007g04980.1
MEIDEAIRESDDRRLKTKYNNAIYVVRRALALYSVEEVALSFNGGKDSTVLLHLLRAGYFLHKREQSHSN
GVLTDHEVAFPIRTIYFESPSAFPEINSFTYETATTYGLQMDIIRLDFKSGLEALLEAKPIRAIFLGVRI
GDPTAVGQEQFSPSSPGWPPFMRVNPILDWSYRDVWAFLLACKIPYCSLYDRGYTSIGSIHDTVPNALLC
VNNSSSSKEKFRPAYLLSDGRLERAGRAKKFSLPVCRPAYPAVSNGMISVDSNKNSTLTATVIGVGDEIL
FGTVEDELGPSLCRKLHSIGWSVSQTAVLRNDVDSVAEEVERWKSTNDVLFIYGGVGPLHSDVTLAGVAK
AFGVRLAPDEEFEEYLRHLIGDHCTGDRNEMAQLPEGITELLHHEKLSVPLIKCQNVFILTATNVTELDK
EWDCLIELTRSSGLLVLMEPFLSKRMTTNLSDDQTRLESAVEALSKKFPAGQISEAN*

>F.x_ananassa v1.0.a1|maker-Fvb6-1-augustus-gene-251.30-mRNA-1
MEIDKAIKECDDRRLKTKYNNAIYVIQRALALYSIEEVAFSFNGGKDSTVLLHLLRAGYFLHKGEQSCTN
GGVQDFPIRTIYFDCPSAFPEINSFTYDAANTYGLQLDIIRSDFKSGLEALLKSNPIRAIFLGVRMGDPT
AVGQEQFSPSSLGWPPFMRVNPILDWSYSYTSIGSIYDTVPNSLLSINNSSSSKEAFRPAYLLSDGRLER
AGRVKKLPHSVGGKKPVVANGLTSMDLPKSSLLMASAIAVGDEILFGTAEDQLGHSLCKKLHSIGWSVSQ
TTVVRNEVDSVAEEVERRQSTNDMVFIYGGVGPLHSDVTLAGVAKAFGVRLAPDEEFEEYLRHLIGDQCT
GDRNEMALLPEGITELLHHEKLMVPLIKCKNVIIFTATNVLELDDEWNCLIELMRSGGELVMAQPFVSKC
LTTNLADLEVAQPVSKLCLEFPDLYIGCYRISRREPLRIYFEGKDQNRIESAKEALCKKFQPGAFSEINL
S*
>E.grandis v2.0|Eucgr.K02925.1.p
MEIDKAVKECDDRRLKTKYANAIYVIQRALALYSVEEVAFSFNGGKDSTVLLHLLRAGHYLSEANQRSPN
GNLKGFPFRTIYFESSSAFSEINSFTHGIATTYALQMEVIHTDFKSGLEDLIKSKSIRAIFLGVRIGDPT
AVGQEQFSPSSPGWPPFMRVNPILDWSYRDVWAFLLISKVPYCCLYDQGYTSIGSIHDTVPNALLRSGDS
ASSEEKFRPAYMLSDGRLERAGRVKKHSSSAHGVSPSSDGMYKLDFNNGSVLTTSIIAVGDEILSGIVED
HLGPILCRKLHSIGWLVSKSVAVRNNVDSVAEEVEQMKSANDIVFICGGVGPLHSDVTLAGIAKSFGVRL
APDEEFEEYLWQLIGNQSKGDRNEMALLPEGITELLHHDKLPVPLIKCQNVIILTATNVTELDQEWDCLI
DLMRSAGQLVVVDPFVSKRLTANLSDVEAAAPLSKLCLEFPDLCIGCYRESRHGPLIISFESKDTARIDS
AIGALRNKFPPEAFSEIN*
>A.hypogaea v1.0|arahy.Tifrunner.gnm1.ann1.ZWC5L9.3
MITSQFAHKTKRWTVLVISVSVLWLTQTTSAIQIPMFLCLRVTMEIDRAIRECSDRRLQTKYNNATYVIQ
RALALYSIEEVAFSFNGGKDSTVLLHLLRAGYFLHKGGQNSANGDLNDFPIRTIYFETPSAFPEINSFTY
DTAATLVKLSLPYTFDYSLNCNCSVDNMEKRYFLLSDNLFLSSYGLQIDTICTDFKSGLEALLKEKPIRA
IFLGVRIGDPTAVGQEQFSPSSPGWPAFMRVNPILDWSYRDVWAFLLTCKVNYCSLYDQGYTSIGSIYDT
VPNSLLSTSDSSDKFKPAYLLADGRLERAGRAKRLSSSNCGQRPVDSNGLSSLDLHKNSMLTASVIAVGD
EFLFGTVEDQLGPNLCRKLHSVGWSVLRLSVVHSNIDSVAEEVEQKKSSDMVFIYGGVGPLHSDVTIAGI
AKAFGVRLAPDEEFEEYLRHIIGDQCIGDRNEMAQLPEGITELLHHDKLSVPLIKCQNVIVLTATNVSEL
EKEWNCLIELTESNDLLTLLEPYVSKQVMTNLSDVEIAQPLSKLCLEFPDLHIGCYRTARYGSLVVSLKG
KDQERLELAMKSLEKKFQPGTFKEMN*
>A.hypochondriacus v2.1|AH022020-RA
MEIDQAIRDSNDQRLKTKYNNAVYVIQRALALYNIEEVAFSFNGGKDSTVLLHLLRAGYYLHKEEQENTN
EGNARDQCKFAIRTIYFESPSAFPEINSFTYETAAAYKLQLDIIREDFKSGLEGLLKATPIRAIFLGVRM
GDPTAVGQEQFSPSSRGWPPFMRVNPILDWSYRDVWAFLLTSKVPYCSLYDQGYTSIGSIYDTVPNKLLC
ISDSSSSKMEYRPAHMLADGRLERAGRARKSTHGSHSNISLSNGTTDVEAPHSCIPTASVIGVGDEILCG
SVEDLIGPSLCRKLHSIGWAVTQFDILRNNIDSIADIVERQKSINDMVFVYGGIGPLHSDATLAGIAKAF
GVRLAPDEEFEEYLTNIVGYHYTGDRNEMARLPEGITELLHHEKLPVPLMKCQNVIIFMATNLMELDNEW
NCLIELSTSNRILMLTKPFASKYLSTNLTEVEVAQPLSTLHIDFPDLYIGSYRKSRHGPLVITLEGKDEG
KVQLAMEALVKKFNPGAFSENV*
>C.citriodora v2.1|Cocit.K0363.1.p
MEIDKAIQECDDRRLKTKYANAIYVIQRTLALYSIGEVAFSFNGGKDSTVLLHLLRAGHYLSEANQGSPN
GNLIGFPFRTIYFESSSAFPEINSFTHGIATTYSLQMEVIHTDFKSGLEDLINSKSIRAIFLGVRIGDPT
AVGQEQFSPSSPGWPPFMRVNPILDWSYRDVWAFLLICKVPYCCLYDQGYTSIGSRHDTVPNSLLRSGDS
VSGEGKFRPAYMLSDGRLERAGRVKKHSSSVHGVSPSSDGMYKLDLNNGSVLTTSIIAVGDEILSGIVED
HLGQMLGRKLHSIGWLVSKSVAVRNNVDSVAEEVERMKSANDIVFICGGVGPLHSDVTLAGIAKSFGVRL
APDEEFEEYLWQLIGDQSKGDRNEMALLPEGITELLHHDKLPVPLIKCQNVIILTATNITELDQEWDCLI
DLMTSNGQLVAVEPFVSKRLTANLSDVEAAAPLSKLCLEFPDLCIGCYRKSRHGPLVISFESKDKARIDS
AIGALRIKFPPEAFSEIN*
>L.usitatissimum v1.0|Lus10013826
MVVSFLQPLDTSIVTVRTVNDQGSLHISEFPERAAQSLARSSSRLLDKQSVAFIAASPCQFSHSLSAPLG
CPVYCGGNRRSFYTLNLLKLPFTWQTTTLESIAVSGKMEIDQAIRESDDRRLKTKYNNAIYVIQRALALY
PIEEVALSFNGGKDSTVLLHLLRAGYYLLKGEQSCSNAGLTSFPLRTIYFESPSAFPEINSFTYDAASRY
GLQLDIVSLDFKTGLENLLKANPIRAIFLGVRIGDPTAVGQEQFSPSSPGWPPFMRVNPILDWSYRDVWA
FLLTCKVQYCSLYDQGYTSIGSIHDTTPNSLLSISDSSGSQEKFKPAYLLSDGRSERAGRVKKMSHPISQ

GSPVLGDSPDVPDSLINRMRIGSAIAVGDEILTSLQLLVLDICLHDFPLPQIDSVAEEVERQKSSNDLVF
IYGGIGPLHSDVTSAGVAKAFGVRLAPDEEFQEYLRHLIGKHCTGDRNEMALLPEGITELLHHEKLDVPL
IKCQNVILLTATNKSELDKEWECLVDFTKSCGLLTVMKPNVSKCLTTHLSDVETAQPLSKLCMQYPDLNI
VCYRKSRAGPLIISFKGKERARIEAAAEALRKKFPEGVFSEN
>A.trichopoda v1.0|evm_27.model.AmTr_v1.0_scaffold00211.1
MDIVRAIQGSQDRRLVTKYNNAIYVIQRALALYSFEEVAFSFNGGKDSTVLLHLLRAGYALQEDRAGCLN
VECSNSKLKHPIRTIYFESPCAFLEINAFTYETAAAYDLQLEIIRLDFKAGLEALLREKPVKAIFLGTRI
GDPNAVGQEQFSPSSPGWPPFMRVNPILDWSYRDVWAFLLTCKVPYCKLYDQGYTSIGSIHDTVPNALLC
ISDSSSDEEKFRPAYLLPDGRLERAGRAKKLSGVPSLSNGLSNIDLHDNNIRTASVIGVGDEILFGKVAD
QLGPSLCRKLHSIGWAVTHVAVVRNDVDSVAEEVERQKAANDMVFVFGGVGPMHSDVSMAGIAKAFGVRL
APDEEFEEYLRHLIGEECSGDRNEMALLPEGITELLHHDTLPVPLIKCHNVISLSSTNEDELDKQWGCLL
EMNASSGLIELMAPFGSKYLCTRLSDVALAQPLSKLCLEFPDLYIGCYRKARTGSGSTIISFMGKNQARV
DSAVDKLCSMFFPSAFSETESG*
>A.comosus v3|Aco015865.1
MEIDRAIRESTDRRLQTKYNNAIYVIQRAFALYKLEEVALSFNGGKDSTVLLHLIRAGYYLHMDKTDFCN
GIQQDGLLNCPIRTIYFESPCAFPEINSFTYETASHYGLPLEIIRSDFKSGLEGLLKEKPTKAIFLGTRI
GDPNAVGQEQFSPSSNGWPPFMRVNPILDWSYRDVWSFILTCKVKYCSLYDQGYTSIGSIYDTVPNTLLC
IADSSSMEENFKPAYMLLDGRLERAGRAKKNSVKSENNSVPSNGLNIVDAHQSASYRASIIIVGDEYLFG
TFEDKLGTALCKKLHDIGWQVSHIAVVQNEIDSVADEVERWKSTDDMVFLLGGLGPLHSDVSVAGVGKAF
GVRLAPDEEFEEYLRQLIGNNYTGDRNEMALLPEGITELLHHKMLPVPLIKCKNVIILAATNHEELDTQW
DCLLELPNINLVRTAPFKSKHLSTMLSEVETAQVLSKLSVDFPDIYIGCHRKSRLGPFIISFVGKDKTRV
ESAAAKLSQSFSEGAFSEADCG*
>M.acuminata v1|GSMUA_Achr10P06640_001
MEIDRAVREGSDRRLQTKYRNAVYVIQRAFALYEFEQVAFSFNGGKDSTVLLHLLRAGYYLHKGKPECSN
GSLSDSVLNCPIRTIYFESPCAFPEINSFTYETATVYGLQLETIHSDFKSGLEALLKEKPTKAIFLGTRI
GDPNAVGQEQFSPSSIGWPPFMRVNPILDWSYRDVWAFILTCKVQYCSLYDQGYTSIGSIYDTVPNALLS
IADSLNAEGTFKPAYMLSDGRLERAGRTKKMHLKCNSTSPNNGVISVTSSGFFTASIVVVGDEILFGTAE
DKLSAALCKKLYGIGWQVTHVAVVQNEIDSVAEEVERRKFTNDLVFLFGGFGPMQSDVSLAGVAKAFGVR
LAPDEEFEEYLRHLIGKHCTGDRNEMALLPEGITELLQHEKLPLPLIKCQNVIILAATNVCELETQWDCL
LELPNTPLVQLAPFVSKHLSSMLSDVEIAQTISKLCLEFPDVYIGCQRKSRVQSLISFVGKDNTRIELAA
GRLCNSFPEGAFSEVNCG*
>L.sativa V8|Lsat_1_v5_gn_1_51600.1
MEIDKAIRESNDQKLKTKYNKAVNVIQRALALYPIEEVAFSFNGGKDSTVLLHLLRAGFFLHQVDNGHSN
GDLSTHASTFPIRTIYFESPSAFPEINSFTYETASSYGVQLDIIRQDFKSGLEALLKSKPTKAIFLGVRI
GDPTAVGQEQFSPSSPGWPPFMRVNPILDWSYRDVWAFLLTCKVPYCSLYDQGYTSIGSVNDTTPNALLC
IKDSGKEKFKPAYMLSDGRLERAGRAKKLPQKVKDGVKGVESHQENTPSASIIAVGDEILSGRVEDELRH
LLCRKIHSIGWNVSHIAVVSSDVDSVAQEVENQKSTSDMVFLYGGVGPLPSDVSIAGVAKALSVCLAPNE
EFEEHLRNLYGEKWSGDSNEMAKLPEGITELLLHEKLPVPLIKCGNVIVFSATNVTEFDQQWDALTNSRL
LATMGSFVSKHFTTSLSDIEVAQSLSKLCFEFPNVNIGCYRKSRTGALTITLEGKDEKMVEAAMEAVSTR
LSSQ*
>D.alata v1.1|Dioal.0542s0009.1.p
MEIDEAIRASSDRRLQTKYNSAAYIIQRAFALYEFEQVAFSFNGGKDSTVLLHLLRAGYFLYKKNTHRPK
GNQMDHVLNCPMRTIYFESPSAFPEINSFTYETASIYDLQLEVIRSDFKSGLEALLEQKPTKAIFLGTRI
GDPNAVGQEQFSPSSIGWPPFMRVNPILDWSYRDVWAFILTSKVQYCSLYDKGYTSIGSIYDTVPNPLLC
TIDSLSNTEKFKPAYLLSDGRLERAGRAKKRNQVSEASIDIRNGPNIIHSHQNGALMASIIAVGDEILFG
NVEDEIAGTLCRKLHAIGWHVSHKSVVRNEIDSVAEEVENRKSTDNLVFLFGGVGPLHSDVSMAGVAKAF
GTRLAPDEEFEEYLRHLIGNHCTGDRNEMALLPEGITELLHHEMLSVPLIKCGNVVILSATNAYEVEKQW
NCLLESANTPLVQLAPFFSKHLSTMLSDVELSETLSRLCLDFPDLNISCHRKARTGPLIISSTGKDQTRV
ELAAKLLSQKYHGAFSEFNFEGV*
>K.laxiflora v1.1|Kalax.0420s0023.1.p
MEIDKAVRESGDRRLQRKYETAVFVIQRALALYSIKEVAFSFNGGKDSTVLLHLLRAGYYLHEQEQGMIS
DGTAEDGPTFPIRTIYFESPSAFPEINSFTYETASKYGLKLDIIRLDFKSGLEALLKSEPIRAIFLGVRN

GDPTAVGQEQFSPSSPGWPPFMRVNPILDWSYRDVWAFLLTCKVPYCSLYDKGYTSIGSIYDTVPNTLLC
TGSSAEGEEHFKPAHRLLDGRMERAGRVKKIVPPVSSCFNTISNGVNSEDEHQHSTPVASIISVGDEILS
GTAVDYLGPLLFRKLHEIGWVVSRSVAVQSDIDSVADEVERRKVDADMVFVYGGVGPLHSDVTLAGVAKA
FGVRMAPHDAFNEYYMSLQGEQCTSNKDEKVQLPEGITELLHHEKLSVPLLKFQNVIVFSSSNVTELEEQ
WDCLLELSKSIGPLKVSEPFVYKHLATNLRDVAISKHLSKLSLQFPDIYVGCFRKSRSGPLVVNLEGKDP
MRIELAAEALCKNFHPGAFTTII*
>P.hallii v3.1|Pahal.5G426400.1.p
MEIDEAVRGCSDRRMRTKYGNAVYVVQRAFALYPFEEVAFSFNGGKDSTVLLHLIRAGYYLYKKDAGDVA
QMDAVKNCPLRTIYFESPCAFPEINSFTYETVSTYGLPLETIHSDFKSGLEGLLKEKPTKAIFIGTRIGD
PNAVGQEQFSPSSPGWPPFMRVNPILDWSYRDVWSFLLTCKVKYCSLYDQGYTSIGSIHDTVPNALLSDS
STEKSFRPAYMLTDGRLERAGRTKKTNHKMEMNSVASNGMNNIEGEQMISRAASIIVVGDEILFGTTEDK
LGAALCKKLHAIGWRVSHVAVVRNEIDSVAEEVERCKSTDDMVFIFGGLGPLHSDVSLAGVAKAFGVRLA
PDEEFEDYLSQLMGNNYSGDRNEMALLPEGITELLHHKKLPLPLIKCRNVITLAATNVDELDTEWDCLLD
TQESGLVQAKPFESKHLGTTLSDVQIAPVLAKLCLEFSDVYIGCHRISRAGPLVVNLTGKDNQRVEAAAE
KLTRSFEGQFSQVDSCK*
>S.bicolor v3.1.1|Sobic.003G118200.1.p
MEIDEAVRGCSDRRLRTKYGNAVYVVQRAFALYPFEEIAFSFNGGKDSTVLLHLIRAGYYLYKKDSGDIA
QMNAVKNCPLRTIYFESPCAFPEINSFTYETVSTYGLPLETIHSDFKSGLEGLLKEKPTKAIFIGTRIGD
PNAVGQEQFSPSSPGWPPFMRVNPILDWSYRDVWSFLLTCKVKYCSLYDQGYTSIGSIHDTVPNALLSDS
STEKSFRPAYMLTDGRLERAGRTKKTNPKVEMNSVASNGMNITEGGQMVSRAASIIVVGDEILFGTTEDN
LGAALCKKLHAIGWRVSHVAVVRNEIDSVAEEVERCKSVDDMVFIFGGLGPLHSDVSLAGVAKAFGVRLA
PDEEFEDYLSQLMGNNYTGDRNEMAQLPEGITELLHHKTLPLPLIKCRNVIALGATNMVELDTEWDCLLD
TQESGLMPTKPFVSKHLSTTVSDVQIAPVLAKLCLEFSDVYIGCHRISRAGPLVVNLTGKDNQRVDAAAE
KLTSSFEGQFSQVDSCK*
>P.virgatum v5.1|Pavir.5KG301100.3.p
MEIDEAVRGCSDRRMRTKYGNAVYVVQRAFALYPFEEIAFSFNGGKDSTVLLHLIRAGYYLYKKDSGDVA
QMDAIKNCPLRTIYFESPCAFPEINSFTYETVSTYGLPLETIHSDFKSGLEGLLKEKPTKAIFIGTRIGD
PNAVGQEQFSPSSPGWPPFMRVNPILDWSYRDVWSFLLTCKVKYCSLYDQGYTSIGSIHDTVPNALLSDS
STEKSFRPAYMLTDGRLERAGRTKKTNHKIEMNSVASNGMNNIEGEQMISRAASIIVVGDEILFGTTEDK
LGAALCKKLHAIGWRVSHVAVVRNEIDSVAEEVERCKSMDDMVFIFGGLGPLHSDVSVAGVAKAFGVRLA
PDEEFEDYLSQLMGNNYTGDRNEMALLPEGITELLHHKKLLVPLIKCRNVITLAATNVHELDTEWGCLLD
NQESGLVQAKPFVSKHLGTTLSDVQIAPVLAKLCLEFSDVYIGCHRISRAGPLVVNLTGKDNQRVEAAAQ
KLTSSFEGQFFQVGNCK*
>S.italica v2.2|Seita.5G055900.1.p
MEIDEAVRGCSDRRLRTKYANAVYVVQRAFALYPFEEVAFSFNGGKDSTVLLHLIRAGYYLYKKDSVDVA
QMDAVKNCPLRTIYFESPCAFPEINSFTYETVSTYGLPLETIQSDFKSGLEGLLKEKPTKAIFIGTRIGD
PNAVGQEQFSPSSPGWPPFMRVNPILDWSYRDVWSFLLICKVKYCSLYDQGYTSIGSIHDTVPNALLSDS
STEKSFRPAYMLTDGRLERAGRTKKTNHKIEMNSVASNGMSNIEGEHMVSRAASIIIVGDEILFGTTEDK
LGTALCKKLHAIGWRVSHVAVVRNEIDSVAEEVERCKSTDDMVFIFGGLGPLHSDVSLAGVAKAFGVRLA
PDEEFEDYLSQLMGNNYTGDRNEMALLPEGITELLHHKTLPLPLIKCRNVITLAATNVDELDTEWDCLLD
TQESGLVQAKPFVSKHLSTTLSDVQIAPVLAKLCLEFSDVYIGCHRISRAGPLVVNLTGKDNQRVEAAAE
KLTSSFEGQFSQVDSCK*
>K.fedtschenkoi v1.1|Kaladp0011s0258.1.p
MEIDKAVRESGDRRLQRKYETAVFVIQRALVLYSIKEVAFSFNGGKDSTVLLHLLRAGYYLHEQEQGMIS
DGTAEDGPTFPIRTIYFESPSAFPEINSFTYETASKYGLKLDIIRLDFKSGLEALLKSEPIRAIFLGVRN
GDPTAVGQEQFSPSSPGWPPFMRVNPILDWSYRDVWAFLLTCKVPYCSLYDKGYTSIGSIYDTVPNTLLC
TGSSAEGEEHFKPAHRLLDGRMERAGRVKKIVPPVSSCFNTISNGVNSEDEHQHSTPVASIISVGDEILS
GTAVDYLGPLLFRKLHEIGWVVSRSVAVQSDIDSVADEVERRKVDADMVFVYGGVGPLHSDVTLAGVAKA
FGVRMAPHDAFNEYYMSLQGEQCTSNKDEKVQLPEGITELLHHEKLSVPLLKFQNVIVFSSSNVTELEEQ
WDCLLELSKSIGPLKVSEPFVYKHLATNLRDVEISKHLSKLSLQFPDIYVGGFRKSRSGPLVVNLEGKDP
MRIELAAEALCKNFHPGAFTTII*
>S.viridis v2.1|Sevir.5G055400.1.p

MEIDEAVRGCSDRRLRTKYANAVYVVQRAFALYPFEEVAFSFNGGKDSTVLLHLIRAGYYLYKKDSVDVA
QMDAVKNCPLRTIYFESPCAFPEINSFTYETVSTYGLPLETIQSDFKSGLEGLLKEKPTKAIFIGTRIGD
PNAVGQEQFSPSSPGWPPFMRVNPILDWSYRDVWSFLLICKVKYCSLYDQGYTSIGSIHDTVPNALLSDS
STEKSFRPAYMLTDGRLERAGRTKKTNHKIEMNSVASNGMNNIEGEHMVSRAASIIIVGDEILFGTTEDK
LGTALCKKLHAIGWRVSHVAVVRNEIDSVAEEVERCKSTDDMVFIFGGLGPLHSDVSLAGVAKAFGVRLA
PDEEFEDYLSQLMGNNYTGDRNEMALLPEGITELLHHKTLPLPLIKCRNVITLAATNVDELDTEWDCLLD
TQESGLVQAKPFVSKHLSTTLSDVQIAPVLAKLCLEFSDVYIGCHRISRAGPLVVNLTGKDNQRVEAAAE
KLTSSFEGQFSPVDSCK*
>N.colorata v1.2|Nycol.B01574.1.p
MEIDEAIKKSKDRRLQAKYKNAVYVIQRAFALYSFDEVAFSFNGGKDSTVLLHLLRAGYALHEEQNNRSK
GIVEGGKSKCPLRTIYFESPCAFPEINAFTYETASTYSLQLEIIRLDFKAGLVGLLKEKPVKAIFLGTRI
GDPNAIGQEQFSPSSCGWPPFMRVNPILDWSYRDVWAFLLTCKVPYCSLYDKGYTSIGSIYDTVPNSLLS
VSDSSSSKDSFRPAYLLADGRLERAGRVKKIAHPTYEKIHVMSNGMNKVGTGSKSLLTASLVAIGDEILF
GAVENKLASVLCRKLHSIGWGVTHAAAVPNDVDAVAEEVELRKSTNDMVFIYGAVGPLHCDVSLAGVAKA
FGVRLAPDEEFEEYLRHLIGDKSSGEKNEMALLPEGITELLHHDMLAVPLIKCQNVIILSATNDDELEKQ
WSCLLELSNTNKILVPMAPFVTKQLQTTLSDIEIAQPLSKICSEFPDVCIGCYRTSRTGQCIITFNGKDE
ERIDSAVEKLCHLFHPSVFYEITCG*
>Z.marina v2.2|Zosma37g00570.1
MEIDKAVRECSDRRLQTKYKNAVYVIERSFILYSFEEVAFSFNGGKDSTVLLHLLRAGYFLHKKNCCDGK
PADSEKRCPIRTIYFENSRAFPEIDSFTYKMAKVYELQLDIISLDFKSGLEDLARKKPIKAIFIGTRIGD
PNAVGQEQFSPSSTGWPPFMRVNPILDWSYRDVWAFLITCKVPYCSLYDQGYTSIGSIHDTTPNELLRVT
DSSNKAEQFKPAYQLSDGRLERAGRRKKTPLISDGADVVVNGVKSSPQPNRSLVASIIGVGDEILYGTFE
HHLIISLCKKLHSIGWKVTHNAIVRNDIDSVAEEVELRKSTNDLVILIGGVGPLHSDVTVAGVAKSFDVR
LAPDEEFEEHLRHLNGIQFTGDRNEMALLPEGITELLHHEMLPVPLIKCRNVIILSATNIEELNAQWGCL
LELPETLLIQLSPFISKHLSTLITDMEISTSLQNLCLRFPDVCIAGHRKSREGPLIISFVGKDIARIEAA
SLDLSQNFKDGTFTETNCE*
>A.officinalis V1.1|evm.model.AsparagusV1_07.1837
MEIDKAVKGSSDRRLQTKYKNATYVIERAFALYEFEEVAFSFNGGKDSTVLLHLLRAGYYLHKEKTKQSN
GNQLDSIPECRIRTIYFETPSAFPEINSFTYETASKKKPIKAIFLGTRIGDPNAVGQEQFSPSSPGWPPF
MRVNPILDWSYRDVWAFLLTCKVQYCSLYDKGYTSIGSIYDTIPNALLSIPDSSSSKGNFKPAYLLADGR
LERAGRAKKSIPKYENIHVLRNGVNDGSQHQISSLSASIISVGDEILHGIVKDTMGAMLCKKLYDIGWLV
AHIAVVPNEIDSVTEEIERRRSTDDMIFLFGGVGPLHSDISVAGVAKAFGVRVAPDEEFEEYLRHLIGDQ
CTGDRNEMALLPEGITELLHHEKLAVPLIKCHNVIILSAANVSELENQWDCLLELPSTPVARMEPFVSKF
LSTKLSDVETAESISNLCFSFPDIHISCHRKSRVGPLIISLTGKDQKRVELAAKELSQKFPEGAFSEVTY
K*
>B.distachyonTek-4 v1|Brdisv1Tek-41007688m.p
MEIDAAVRASSDGRLRTKYGSAVYVVQRAFALYPFEEIAFSFNGGKDSTVLLHLLRAGYYLHKTSSGDGA
QTDTIQNCPLRTIYFETPCAFPEINSFTYETVSTYGLPLETIRSDFKSGLEGLLKEKPTKAIFIGTRIGD
PNAVGQEQFSPSSPGWPPFMRVNPILDWSYRDVWSFLLTCKVKYCSLYDEGYTSIGSIYDTVPNALLSDS
STGKSFRPAYMLSDGRLERAGRMKKTSSKTGTNSVASNGMNNAEGEQMISRSASIIVVGDEILFGAVEDE
FGAALCKKLNEIGWRVSHVAVVHNEIDSVAEEVGRCKSTDDTVFLLGGLGPLHSDVSLAGVAKAFGVRLA
PDEEFEEHLSQLIGNSYTGDRNEMALLPEGITELLHHKTLPLPLIKCKNVIVLAATNVDELAMEWNCLLD
TQESGLVRAKPFVSKHLRTLLPDVKIAPVVAKLCLEFSDVYIGSHRISRTGPLVVSLVGKDNQRVEGAAE
KLASSFEGQFSQVDSCK*
>B.stacei v1.1|Brast01G309700.1.p
MEIDAAVRASSDGRLRTKYGSAVYVVQRAFALYPFEEIAFSFNGGKDSTVLLHLIRAGYYLHKTSSGDGA
QTDTIQNCPLRTIYFETPCAFPEINSFTYETVSTYGLPLETIRSDFKSGLEGLLKEKPTKAIFIGTRIGD
PNAVGQEQFSPSSPGWPPFMRVNPILDWSYRDVWSFLLTCKVKYCSLYDEGYTSIGSIYDTVPNALLSDS
STGKSFRPAYMLSDGRLERAGRMKKASNKTGTNSVASNGMNNAEGEQMISRSASIIVVGDEILFGTVDDE
FGAALCKKLNEIGWRVSHVAVVHNEIDSVAEEVGRCKSTDDTVFLLGGLGPLHSDVSLAGVAKAFGVRLA
PDEEFEEHLSQLIGNSYTGDRNEMALLPEGITELLHHKTLPLPLIKCKNVIVLAATNVDELAMEWNCLLD

TQESGLVRAKPFVSKHLSTLLPDVKIAPVVTKLCLEFSDVYIGSHRISRTGPLVVSLVGKDNQRVEDAAE
KLASSFEGQFSQVDSCK*
>B.hybridum v1.1|Brahy.S01G0323700.1.p
MEIDAAVRASSDGRLRTKYGSAVYVVQRAFALYPFEEIAFSFNGGKDSTVLLHLIRAGYYLHKTSSGDGA
QTDTIQNCPLRTIYFETPCAFPEINSFTYETVSTYGLPLETIRSDFKSGLEGLLKEKPTKAIFIGTRIGD
PNAVGQEQFSPSSPGWPPFMRVNPILDWSYRDVWSFLLTCKVKYCSLYDEGYTSIGSIYDTVPNALLSDS
STGKSFRPAYMLSDGRLERAGRMKKASNKTGTNSVASNGMNNAEGEQMISRSASIIVVGDEILFGTVDDE
FGAALCKKLNEIGWRVSHVAVVHNEIDSVAEEVGRCKSTDDTVFLLGGLGPLHSDVSLAGVAKAFGVRLA
PDEEFEEHLSQLIGNSYTGDRNEMALLPEGITELLHHKTLPLPLIKCKNVIVLAATNVDELAMEWNCLLD
TQESGLVRAKPFVSKHLSTLLPDVKIAPVVTKLCLEFSDVYIGSHRISRTGPLVVSLVGKDNQRVEDAAE
KLASSFEGQFSQVDSCK*
>H.vulgare r1|HORVU3Hr1G035900.1
MEIDAAVRASSDGRLRTKYDNAVYVVQRAFALYPFEEIAFSFNGGKDSTVLLHLIRAGYYLHKTSSGDEA
QINTVQNCPLRTIYFETPCAFPEINSFTYETVSTYGLPLETIRSDFKSGLEGLLKEKPTKAIFIGTRIGD
PNAVGQEQFSPSSPGWPPFMRVNPILDWSYRDVWSFLLTCKVKYCSLYDEGYTSIGSIYDTVPNALLSDS
STGKSFRPAYMLSDGRLERAGRAKKTSNKTETNSVASNGMNNAEGEQMISRSASVIVVGDEILFGTVGDK
VGAALCKKLHEIGWRVSHVTVVHNEIDSVAEEVEQCKSTDDVVFILGGLGPLHSDVSLAGVAKAFGVRLA
PDEEFEEHLSQLIGNSYIGDRNEMALLPEGITELLHHKMLPLPLIKCKNVIILSATNVDELDMEWNCLLD
TQESGLVKTKPFVSKHLSTLLPDVKIAAVVAKLCLEFSDVYIGSYRISRMGPLVVSLIGKDNQRVQEAAL
KLSGSFEGQFSQVDGCK*
>B.sylvaticum v1.1|Brasy1G390900.1.p
MEIDAAVRASSDGRLRTKYGSAVYVVQRAFALYPFEEIAFSFNGGKDSTVLLHLIRAGYYLHKTSSGDGA
QTDTIQNCPLRTIYFETPCAFPEINSFTYETVSTYGLPLETIRSDFKSGLEGLLKEKPTKAIFIGTRIGD
PNAVGQEQFSPSSPGWPPFMRVNPILDWSYRDVWSFLLTCKVKYCSLYDEGYTSIGSIYDTVPNALLSDS
STGKSFRPAYMLSDGRLERAGRTKKTSNKTGTNSVASNGMNNAEGEQMISRSASIIVVGDEILFGTVEDE
FGAALCKKLNEIGWRVSHVAVVHNEIDSVAEEVGRCKSTDDTVFLLGGLGPLHSDVSLAGVAKAFGVRLA
PDEEFEEHLSQLIGNSYTGDRNEMALLPEGITELLHHKTLPLPLIKCKNVIVLAATNVDELAMEWNCLLD
TQESGLVRAKPFVSKHLSTLLPDVKIAPVVAKLCLEFSDVYIGSHRISRTGPLVVSLVGKDNQRVGEAAE
KLASSFEGQFSQVDSCK*
>S.polyrhiza v2|Spipo9G0006400
MEIEKAINGCSDRRLQTKYKNATYVIRRTFALYSFEEIAFSFNGGKDSTVLLHLLRAGFFLHNKDRGFVG
SETDSKKECAIRTIYFESPCAFPEINSFTYDIATTYGLQLEIIHSDFKSGLEDLLKKKSTKAILLGTRIG
DPNAVGQEQFSPSSFGWPPFMRVNPILDWSYRDVWAFLLTCKVEYCSLYDKGYTSIGSIHDTVPNALLSF
TDSDNGEHFRPAYLLSDGRLERAGRARKYPSKYDTVLTVSNGKSMDEFSSKTLSASIIAVGDEILFGLFE
DQMGVSLCKKLHAIGWQVTHQSVVRNEVDSVAEEVEKRMSTNDMVFIFGGLGPMHSDVSVAGLAKAFGVR
LAADEEFEEYLRHLIGDHCTGDRNEMALLPEGITELLHNDVLPVPLIKCRNVIVLTSTNVAELDKEWDCL
LTLPNTPLVQMAPFSSKHLSTMLSDVEAAQTVARLCLDFPDIYIVIVDLELDP*
>T.intermedium v2.1|Thint.08G0266700.1.p
MEIDAAVRASSDSRLRTKYDNAVYVVQRAFALYPFEEIAFSFNGGKDSTVLLHLIRAGYYLHKTSCGDEA
QVNTVQNCPLRTIYFETPCAFPEINSFTYETVSTYGLPLETIRSDFKSGLEGLLKEKSTKAIFIGTRIGD
PNAVGQEQFSPSSPGWPPFMRVNPILDWSYRDVWSFLLTCKVKYCSLYDEGYTSIGSIYDTVPNALLSDS
STGKSFRPAYMLSDGRLERAGRAKKTSNKTETNSVASNGMNNAEGEQMISRSASVIVVGDEILFGTVEDK
FGAALCKKLHEIGWRVSHVRVVHNEIDSVAEEVEQCKSTDDVVFIFGGLGPLHSDVSLAGVAKAFGVRLA
PDEEFEEHLSQLIGNSYIGDRNEMALLPEGITELLHHKMLPLPLIKCKNVIILSATNVDELDMEWNCLLD
TQESGLLRTKPFVSKHLSTLLPDVKIAPVVAKLCLEFSDVYIGSHRISRTGPLVVSLIGKDNQRVEGAAA
KLSGSFEGLFSQVDGCK*
>T.aestivum v2.2|Traes_3B_30DFEE86D.1
MKYPHEASLASHPSLPPSSNADESRLLSSPQLLSSQSLLLPPQVASPGMEIDAAVQASSDGRLRTKYDNA
VYVVQRAFALYPFEEIAFSFNGGKDSTVLLHLIRAGYYLHKTSSGDEAQINTVQNCPLRTIYFETPCAFP
EINSFTYETVSTYGLPLETIRSDFKSGLEGLLKEKSTKAIFIGTRIGDPNAVGQEQFSPSSPGWPPFMRV
NPILDWSYRDVWSFLLTCKVKYCSLYDEGYTSIGSIYDTVPNALLSDSSTGNSFRPAYMLSDGRLERAGR
AKKTSNKTETNSVASNGMNNAEGEQMISRSASVIVVGDEILFGTVEDKFGAALCKKLHEIGWRVSHVTVV

```
HNEIDSVAEEVERCKSTDDVVFIFGGLGPLHSDVSLAGVAKAFGVHLAPDEEFEEHLSQLIGNSYIGNRN
EMALLPEGITELLHHKMLPLPLIKCKNVIILSATNVDELDMEWNCLLDTQESGLLRTKPFVSKHLSTLLP
DVKIAPVVAKLCLEFSDVYIGSHRISRTGPLVVSLIGKDYQRVEGAAAKLSGSFEGLFSQVDSCK*
>M.sinensis v7.1|Misin06G099200.1.p
MNAVKNCPLRTIYFESPCAFPEINSFTYETVSAYGLPLETIHSDFKSGLEGLLKEKPTKAIFIGTRIGDP
NAVGQEQFSPSSPGWPPFMRVNPILDWSYRDVWSFLLTCKVKYCSLYDQGYTSIGSIHDTVPNALLSDSS
TEKSFRPAYMLTDGRLERAGRTKKTNPKVEMNSVASNGMNYTEGGQMISRAASIIVVGDEILFGTTKDNL
GAALCKKLHAIGWRVSHVAVVHNEIDSVAEEVERCKSTDDMVFIFGGLGPLHSDVSMAGVAKAFGVRLAP
DEEFEDYLSQLMGNNYTGDRNEMALLPEGITELLHHKTLPLPLIKCRNVIALAATNMDELETEWDCLLDT
QESGLMPAKPFVSKHLSTTLSDVQIAPVLAKLCLEFSDVYIGCHRISRAGPLVVNLTGKDNQRVDAAAEK
LTSSFEGQFSQVDSCK*
>S.fallax v1.1|Sphfalx02G007000.1.p
MEICREVLDSGDKRLQAKFKHALNVIDRTLALYKFEEVAFSFNGGKDSTVLLHLLRAGYAVAEERLVVSS
LEVKAAKSHPIRTIYFESSYAFPEIDLFTLETAKLYNLEMEIIRLDFKTGIEALLREKPIKAIFLGTRIG
DPNAAGQEEFAPSSAGWPPFMRVNPILNWSYRDVWAFLLACKVPYCKLYDQGYTSIGSIHDTVPNCALCV
SSDDTSTLDTSPDGDLEEPTQSKDKYRPAYLLRDGRLERAGRLKKVSKSKSENGIASELPGQNLFAASIM
VVGDEILRGEVEDQVGKYLSKALYSIGWAVTRCTVLPNDIDSISEEVEQRARVSDIVLITGGVGPIHSDV
TLAGVAKAFGVRLASDEEFAEDLRQPLGPNFSGDQNKMAKLPEGITELLHLQDCSVPVIKCRNVFVLLGP
TVEEVAEQWKSLVELSKERKLFHVSQGFTCVRLCTSLPDVEIAGPLSEIDLDFPDVTFGCYRESLQSQLV
KKGAQSPGGLILSLVGKNGRRVACAARALHAAFAPGSLSELPEG*
>O.thomaeum v1.0|Oropetium_20150105_09721A
MEIERAMRGSGDARLRAKYDAAVHVVRRAFALYPWLQFISSYSNAKKEVYFILLGLNGTSLCRFEELAFS
FNGGKDSTVLLHLLRAGYYLHRTDSGDATNTDRATTGIQNCRMRTIYFEEPNAFPEIDSFTYDTASTYGL
PLEIIRTDFKSGLEALLKEIPTKGIFLGTRNGDPNAVGQQQFSPSSPGWPPFMRINPILDWSYRFYFLNF
TSYHVTKLRKRFAFSLASHHAICFDSDVWSFLLTCNVKYCSLYDQGYTSIGSIHNTVPNEELKDGSGGFR
PAYMLSDETLERAGRGAKKYATAPNNAVVVRQTSRRMEIDEAVRGSSDRRLRTKYGNAVYVVQRAFALYP
FEEVAFSFNGGKDSTVLLHLIRAGYYLYKRDSGEEAQNDAIENCPVRTIYFESPCAFPEINSFTYETVSN
YGLPLETIRSDFKSGLEGLLKEKPTKAIFIGTRIGDPNAVGQEQFSPSSPGWPPFMRGCLVFSFDMYTSI
GSIHDTVPNALLSDSSCGKSFRPAYMLSDGRLERAGRAKKANNNMEVNFVASNGMNVTEEEEMVSRSASI
VIVGDEILFGTAEDKLGSALCNKLHAIGWRVSHIVVVRNEVDSVAEEVDRCISTDDMVFTFGGLGPLHSD
VVLAGVAKAFGVRLAPDEEFEDYLSQMMGNDYTGDRNEMALLPEGITELLHHKLLPLPLIKCRNVITLAA
TNAHELEIEWDCLLDTQESGLVRAKPFVSKHLRTALSDVKIAAVLAKLCLEFSDVYIGSHRVSRSGPLVV
NLTGKDTWRVEAVAEKLMSSFEGQFSELY*
>T.pratense v2|Tp57577_TGAC_v2_mRNA33919
MYSLFLRCTSKHYQLEIMSYHIYLLIFICRDVWAFLLTCKVNYCSLYDQGYTSIGSIYDTVPNSLLSISN
SSSKFKPAYLLADGRLERAGRVKRTSSAGGQLPADSNGLDLHKNSTLTASIIAVGDEILFGIVEDQLGPY
LCRKLGSVGWSVLQYSVVHNNIDSVAEEVERQKSRTDMVFIYGGVGPLHSDVTLAGIAKAFDVRLAPDEE
FEEYLRHIISDQCIGDRNEMAQLPEGITELLHHDNLTVPLMKCQNVIILSATDISEMENQWDCLIELTKS
SDLLTLLEPYISKHVTTNLSDVEVAPRLSKLCLEFPDLCIGCYRKSRDGSVIISFKGKDLTRLESAISAL
QKKFKSGAFTETK*
>B.vulgaris EL10_1.0|EL10Ac7g16646.1
MEIDKAIGDCNDRRLKTKYNTAVYVIQRALALYNIEEVAFSFNGGKDSTVLLHLLRAGHYLHQQKQAKHD
EDPAHDHCKFAIRTIYFESPSAFPEINSFTYETATTYNLQLDIIRQDFKSGLEGLLKTKPIRAIFLGVRI
GDPTAVGQEQFSPSSPGWPPFMRVNPILDWSYRDVWAFLLNSKVPYCSLYDQGYTSVGSIYDTVPNKLLS
VSNPSSGKVEYRPAYMLADGRSERAGRARKSTYGADSSISLSNGASNVESLQTCIPTASVIGVGDEILLG
TVEDLVGPSLCRRLHSIGWQVTQFDVLRSDVFRGPAGGVVLVLAPSVGGTNRDLDFGSVASKNIFPVKKW
LWRFSPEPGPIFKSDFDFNSCDDILVKMVIEILQIDAVAESVERQKSTNDMVFIYGGIGPLHSDATLAGI
AKAFGVRLAPDEEFEEYLTNIVGYQHTGDRNEMAQLPEGITELLHHENLPVPLMKCQNVIVLMATNPLEL
DKEWNCLIELTASSGLLTLTKPFSSKYLTTILSDVEVAQPLSALRLDFPDLYIGCYRKSRSGPLVITLLG
KDQGKVQLATEALMKKFHPGAFSGTN*
>A.coerulea v3.1|Aqcoe3G160400.1.p
```

MEIDKAMRESDDQRLKTMYNNAINVIQRALSLYKFEEVAFSFNGGKDSTVLLHLLRAGYFLHIEKPEYPN
DNPANCNVKCPIRTIYFETPSTFPEINSFTHETATTYGLQMETIRLDFKSGLEALLKEKPTKAIFLGTRI
GDPNAVGQEQFSPSSPGWPPFMRVNPILDWSYRDVWAFILTCKIQYCSLYDKGYTSIGSIYDTVPNSFLC
TNDSSSNKDYFKPAYLLADGRLERAGRAKRYSPLASEGGPVASNGPNSVESHQNSTLTASVITVGDEILW
QMLYELHMDDN*
>S.cerevisiae FPY1
MVKVTAACIIIGDEVLNGKVVDTNSTFFAKYCFDHGIQLKEIATIGDDETQIVDTVRRLVKNYDFIISTG
GIGPTHDDITYECMAKSFNLPCELDEECKERMRHKSDPEARLDADALKAHYQMATMPKGTNVKNYYVCDD
LWVPICSISHKMYILPGIPQLFARMLKAFTPTLKKIYNLDKDPREYVRYFVRTHLTESQISKELKLIQDE
STKVSEAIKIGSYPHFGMGFNTVSILGEKKDDSYLKSIVNRVVNNLEGEVISSELENKFSNQES*
>A.thaliana At5g03430
MEIDKAIGESDDKRLKTKYNNAIFVIKRALALYSIEEVAFSFNGGKDSTVLLHLLRAGYF
LHKKEQTCSNGGLSSFPVRTIYFESPSAFTEINAFTYDAAQTYNLQLDIIRQDFKSGLEA
LLKANPIRAIFLGVRIGDPTAVGQEQFSPSSPGWPPFMRVNPILDWSYRDVWAFLLTCKV
KYCSLYDQGYTSIGSIHDTVPNSLLSVNDTSSKEKFKPAYLLSDGRLERAGRVKKIASLK
KDVDTESQKHEVLLASVIAVGDEILSGTVEDQLGLSLCKKLTSVGWSVQQTTVLRNDIDS
VSEEVDRQRSTSDMVFIYGGVGPLHSDVTLAGVAKAFGVRLAPDEEFEEYLRHLISDQCT
GDRNEMAQLPEGITELLHHEKLSVPLIKCRNVIVLAATNTEELEKEWECLTELTKLGGGS
LIEYSSRRLMTSLTDVEVAEPLSKLGLEFPDIYLGCYRKSRQGPIIICLTGKDNARMDSA
AQALRKKFKKDVFVEIK*
>G.max XP_003554393
MEIDKAIRECDDRRLQTKYNNATYVVQRALALYSIEEVAFSFNGGKDSTVLLHILRAGYFLHKKGQNSAN
GDLKDFPIRTIYFESPCAFPEINSFTYDTAATYGLQIDTISLDFKSGLEALLKEKPIRAIFLGVRIGDPT
AVGQEQFSPSSPGWPPFMRVNPILDWSYRDVWAFLLTCKVNYCSLYDQGYTSIGSIYDTVPNSLLCISNS
SNKFKPAYLLADGRLERAGRAKRPSTSTGGQHPAESNGLTSQDSLKNSMLTASIIAVGDEILFGIVEDQL
GPYLCRKLHCIGWSVFQHSVVHNNIDSVAEEVERQKSKSDMVFIYGGVGPLHSDVSIAGIAKAFGVRLAP
DEEFEEYLRHIIGDQCTGDRNEMAQLPEGITELWHHDKLSVPLIKCENVIILSATNVPELEKQWDCWIEL
AKSSDLLALLEPYVSKDVTTNLSDVEIAQPLSKLCLEFPDLYIGCYRKARYGSLIVSFKGKDLTRIESAI
KALHKKFQPSAFVEMN*
>N.tabacum XP_016463627.1
MEIDKAIRECDDGRLKTKYNNAIYVIKRALALYSVQEVALSFNGGKDSTVLLHLLRAGCFLHEAEENNLR
GDAADGGKTFPIRTIYFESPSAFPEINSFTYEAAATYNIQMDIIRLDFKSGLEALLKANPIRAIFLGVRI
GDPTAVGQEQFSPSSPGWPPFMRVNPILDWSYRDVWAFLLVCKVQYCSLYDQGYTSIGSIHDTVRNALLC
IRNSDNSEEKFKPAYLLADGRLERAGRVKKNPSSVCGKLSSISNGGKMENLNSGSMLTASIISVGDEILF
GTVEDKLGSMLCKKLHSIGWAVSRVAVTRNDIDSVAEEVERRKSTDDMVLIFGGIGPLHSDVTVAGVAKA
FGVRMAPDEEFEEHLRHLIGEKCSGDKNEMALLPEGITELLHHEQLPVPLIKCHNVIILTATNVVELDRQ
WDCLIELAKSNGILVLMDPFVSKCFATTLSDVEVAQPLSKLCAQFPDLYIGGYRRSREGPVVITFEGKDL
SRIEAASQSLCQKFHAGAFSEIE*
>O.sativa XP_015621933.1
MEIDRAVRGSSDRRLRTKYDNAVYVVQRAFALYPFEEVAFSFNGGKDSTVLLHLLRAGYYLHKSSSDGEV
EMNTIQNCPVRTIYFESPCAFPEINSFTYETVSTYGLPLETIRSDFKSGLEGLLKERPTKAIFIGTRIGD
PNAVGQEQFSPSSPGWPPFMRVNPILDWSYRDVWSFLLTCKVKYCSLYDQGYTSIGSIYDTVPNALLCDS
TTGKSFRPAYMLSDGRLERAGRTKKNISSVSSNGTNSTEVEQTISRSASIIVVGDEILFGTVEDKLGAGL
CKKLHAIGWRVSHVAVVSNEIDSVAEEVERCKSTDDMVFLVGGLGPLHSDISLAGVAKAFGVRLAPDEEF
EEYLSQLIGDNYTGDRNEMALLPEGITELLHHKMLPLPLIKCKNVVILAATNVDELETEWGCLLDTQESG
LVMAKSFVSKHLCTSLLDVKIAPVVAKLCIDFSDVYIGCYRISRSGPLVVSFIGKDNQRVEAAAEKLTNS
FEGQFSQVDSCK*
>Z.mays AQK89528.1
MEIDQAVRGCSDRRMRTKYSNAVYVVQRAFALYPFEEVAFSFNGGKDSTVLLHLIRAGYYLYKKDSGDVA
QTDAVKNCPLRTIYFESPCAFPEINSFTYEIVSTYGLPLETIHSDFKSGLEGLLKEKPTKAIFIGTRIGD
PNAVGQEQFSPSSPGWPPFMRVNPILDWSYRDVWSFLITCKVKYCSLYDQGYTSIGSIHDTVPNALLSDS
SSEKSFRPAYMLTDGRLERAGRTKKINPKVEMNSAASNGINDTEGGRMISRAASIIVVGDEILFGTTEDN

LGAALCKKLHAIGWRVSHVAVVRNEIDSVAEEVERCKSTDDMVFIFGGLGPLHSDVSLAGVAKAFGVRLA
PDEEFEDYLSQLMGNNYTGDRNEMAQLPEGITELLHHKTLPLPLIKCRNVIALAATNMDELDTEWDCLLG
TQESGLMPSKPFVSKHLSTTLSDVQIASVLAKLCLEFSDVYIGCHRISRAGPLVVNLTGKVDDEFSVEFR
*
>S.magellanicum KAH9570159.1
MEICREVLDSGDKRLQAKFKHALNVIDRTLALYKFEEVAFSFNGGKDSTVLLHLLRAGYAVAEERLVVSS
AEVKAAKPHPIRTIYFESSHAFPEIDLFTLETAKLYNLEMEIIRLDFKTGIEALLREKPIKAIFLGTRIG
DPNAAGQEEFAPSSAGWPPFMRVNPILNWSYRDVWAFLLACKVPYCKLYDQGYTSIGSIHDTVPNCALCI
SSDDTSTLDTSLDGDFEEPAQSKDKYRPAYLLRDGRLERAGRLKKVSRSKSENGIASELSGQNLFAASIL
VVGDEILRGEVEDQVGKYLSKALYSIGWAVTRCTVLPNDIDSISEEVEQRASVSDIVLITGGVGPIHSDV
TLAGVAKAFGVRLASDEEFAEDLRQPLGPNFSGDQNKMAKLPEGITELLHLQDCSVPVIKCRNVFVLLGP
TVEEVAEQWKSLVELSKERKLFHVSQCFTCVRLCTSLPDVEIAGPLSEIDLDFPDVTFGCYRESLQSQLV
KKGAQSPGGLILSLVGKNGRRVAYAARALHAAFPPGSLSELPEG*
>C.richardii KAH7289897.1
MEIWKAVADSGDKRLQAKCKHALYVIERTLALYKFDEVAFSFNGGKDSTVLLHLLRAGYAAAEAKQGFNN
GSYSENQKQHPIRAIYFESPDVFPEIDVFTLETASLYKLEMEIIRLDFKSGLEALLREKPIKAIFLGTRI
GDPNAVGQEQFSPSSTGWPPFMRVNPILDWSYRDVWAFLLACKVPYCKLYDQGYTSIGSIHDTIPNGALC
IESCQITNEEQIEPGHIKYRPAYMLRDGRLERAGRMKRSNKMERKKSVTANGDADNMPCANSLFAASIIV
VGDEILQGDVEDNLGPFLNKKLHSFGWAVTRRVIIPNDIDAISEEVEQRAEVNDLVFVVGGVGSLHSDVS
LAGVAKAFGVRLAPDEEFEEFLRQQVGELCSGDQNEMAKLPEGITELWHHKDLAVPVIKCRNVFVLSGTT
VDELDLQWKCLVELSKGTDIIEGKQQFTSVRLQTTISELELAGPFSRIDKDFPDISIGCYRRSRQVKMRT
QNSDRKSTLVLTFVGKDVDRLRMAVASLSSVFPEGTFLEID*
>M.polymorpha PTQ37944.1
MDIWNSVAECGDKRLQAKFKLSMNVIDRTLSLYGCEEVAFSFNGGKDSTVLLHLLRAGYAVAESQIAPPA
CTEDVRSRTQHRIRTIYFESPDVFPEIDSFTLDTAKAYDLELELIRLDFKSGLVELQKKRPMKAVFLGTR
IGDPNAVGQEQFSPSSAGWPAFMRVNPILDWSYRDVWAFLLACEVPYCSLYDQGYTSIGSIHDTLPNAAL
CISDSSSDDNSAGTAEGKKLPAPDRKFRPAYLLRDGRLERAGRLKNFASVKVKSRDSLRENGDINGLNGM
NGLFAAAILVVGDEILRGDVDDILGMHLSKELYSIGWTVTRRAVLPNDIDAISEEVEHRASDCDIVIMTG
GVGTMHCDVTLAGVAKAFGARLAPDEELEEDLRQHYGENCPGDENKMARLPEGITELLHHKDAAVPVIKC
RNVYVLSGVTTSELALQWRCLLESAKEKDLFHVQQPFTSIRLRTSLPEVELAGPLARFDSEFPDLTIGCY
RESCQFQAIKGGDSRNQGQLIISVVGKEPSRVNSAAKTLANAFPEGVFSEMSKG*
>C.reinhardtii XP_001693086.1
MADILDSLESIPDPVVLEKSKKAIACLRRTCALYPLEKIAFSFNGGKDSTVLLHLLRAAVAQQEAAKGSA
GLGGMLSFYFRQKDDFAEVKAFVEGADKAYGLDVEYLSEQDFKKGLCEYLERTGVMAIVLGTRRGDPNAG
GQDVFCPSSEGWPAFMRVNPILDWTYHDVWVFLRATRVAYCSLYDHGYTSLGGVGNTLPNNTLRRPDGSY
EPAYHLADGRLERVGRVHAAPAKAPAAAMVQAAHDAHAAQPQPGQAAGSGGSAGGAASACGASQGGVAVP
VHHDSGEVGAVQANGEAPAAAPAPAPAPARSGGGAPEAVSELTRSAAIVVVGDELLSGKVDDVNARFLCR
ELRSLGWQVLRVVFVPDAVDDIASVVRALAATAEVVITAGGIGPTLDDVTMQGIALAVGQPLVRLPGMVE
RMASYFGGEAVLTPAHLKMAEGPQHADLIDYKLADGNASKFPILKINNIYVLPGVPSLLVQKWGALKERL
RDDALAPFRNATLRLSLTDETTIAPALERVASTHGLDVAVGSYPVDNGIIVTLDSKNTRALQAAVADITE
LLPGEAVVALERDVDSLQ*
>C.sorokiniana PRW59554.1
MADVLQAIESCEDSRLRARCLKTCAVVSRALDLYGTRGLAFSFNGGKDSTVLLHIIRATLAQRQRQHEQA
AGTPWAGDDLPLGGVLTFFFHHDTDFPEVLEFTHTTSKEYGLGMEILTGDFKQGLSQLLAQTHVQAIVLG
TRRGDPNAADQETFCPSSPGWPPFMRVNPILDWSYHDVWSFLQLAKVPYCSLYDQGYTSLGSVHNTVPNS
ALRLEDGSFAPAHMLPDARLERAGRQSKVERQVSTVGGERRTAGLLIIGDEILSAKVEDVNTRFLCSELR
STGWTVEKAVVVRDDVEAICREVRALSAAHDIVITAGGLGPTLDDVTMQAMADALDQQLALHPQLESRIR
AYFGANTTKAHLKMAEAPTGSEVHLIEYRLEGGALSPFPLLRCRNVYVLPGIPALLQAKWRAVEEHLQSE
APPAAPFRTALLRLRLSDETQVAAALEQVAAAAGEEVTLGSYPVSDQVDDAGIVLSLESRSSEALAAACE
RLKGLLPLGTLLSEHRDSSAINTPSVSPAVGAGANGGLAPAAS*
>D.rerio AAH80254.1
MAQNCNTSSTQKNGSATAAILIIGDEILKGHTVDTNSAFLCRGLRKLGITVERITVVPDVQEVIAKEVSQ

```
LSSTVTHLITSGGIGPTHDDVTFESVAMAFGEELYAHPEMTKLVEGFFGTVTSDSAPMKLAMVPASAKLN
FGIDPQTGQRNRFPLVSVHNVYIFPGIPSLLEKSFNGLSHLFSGSGTTFHTREVFVNADETEIAQSLSKL
QAGWGKRVSLGSYPDWLSNYHRVRLVLDTDSVEEVERARTQLIEELPKGSVVPLVTDPISVAAQEVYSLS
KSETQLGKKVAAALGTIEMALDKYSVNEICVGFNGGKDCTALLHLYYAALKRRYPDGKDRLKALYIRIVS
PFPEMERFLQDTIKRYDLELFSVEGSIRQALNEVKERRPDLRAVLMGTRRTDPYSHTLTPFCPTDPGWPD
YMRVNPLLEWTYHDIWSFLRTLYVPYCILYDKGYTSLGSMDNSYRNPSLKMVDERGATRYKPAYMLENEE
EERNSRE*
>C.elegans NP_001022287.1
MPQRKTAAILVIGDEILKGTTRDTNSHFLCKRLHKLGVNIRKISVIGDDISEISREVQSASGAYDYVITS
GGVGPTHDDKTYLGLAHAFTDQMQFSDEIRQAVNRFLPTYTAKKRAEGVGEGLEEAVRLATEKLCTIPKM
SQLLWGTQKINGSLSTFPVVRISNVVALPGVPKFCERAFDELQDQLFPIEERQSLCFETLYTDLDEFDFS
KKLTDLAAQFEDRNVQIGSYPELKNKFFKTKLTIETESSETMEAVVTSLRELLAGHIVYYDSHAWLDIVT
KWKAFKKRKASENQIEFIQKLNEAESIVEEIVEKYPLEQIALSFNGGKDCTVLLHLLRLKVDEKYGPSTP
IQGFHIMVEDQFPEATQFIIDAAKFYNIQVLEFPGPLKTGLAALKKTRPSIIPVLMGSRATDPNGKYMKT
PVEWTDSDWPQVLRVCPILNWTYTDVWHMLRGLCVPYCKLYDQGYTSLGGRDNTVKHPALRIVSSDGREH
YLPAYKLHNDAEERCNRSNI*
>H.sapiens NP_958800.1
MTSRASELSPGRSVTAGIIIVGDEILKGHTQDTNTFFLCRTLRSLGVQVCRVSVVPDEVATIAAEVTSFS
NRFTHVLTAGGIGPTHDDVTFEAVAQAFGDELKPHPKLEAATKALGGEGWEKLSLVPSSARLHYGTDPCT
GQPFRFPLVSVRNVYLFPGIPELLRRVLEGMKGLFQNPAVQFHSKELYVAADEASIAPILAEAQAHFGRR
LGLGSYPDWGSNYYQVKLTLDSEEEGPLEECLAYLTARLPQGSLVPYMPNAVEQASEAVYKLAESGSSLG
KKVAGALQTIETSLAQYSLTQLCVGFNGGKDCTALLHLFHAAVQRKLPDVPNPLQILYIRSISPFPELEQ
FLQDTIKRYNLQMLEAEGSMKQALGELQARHPQLEAVLMGTRRTDPYSCSLCPFSPTDPGWPAFMRINPL
LDWTYRDIWDFLRQLFVPYCILYDRGYTSLGSRENTVRNPALKCLSPGGHPTYRPAYLLENEEEERNSRT
```
