# Supplementary material for: A higher plant FAD synthetase is fused to an inactivated FAD pyrophosphatase
Source: J Biol Chem. 2022 Oct 20;298(12):102626. doi: 10.1016/j.jbc.2022.102626 (PMC9678776; doi:10.1016/j.jbc.2022.102626)
Supplement: Supplemental Figures S1–S8 [file mmc2.pdf]

Supporting information for:

A higher plant FAD synthetase is fused to an inactivated FAD pyrophosphatase

Joseph Lynch and Sanja Roje

Contents:

Support figures S1-S8

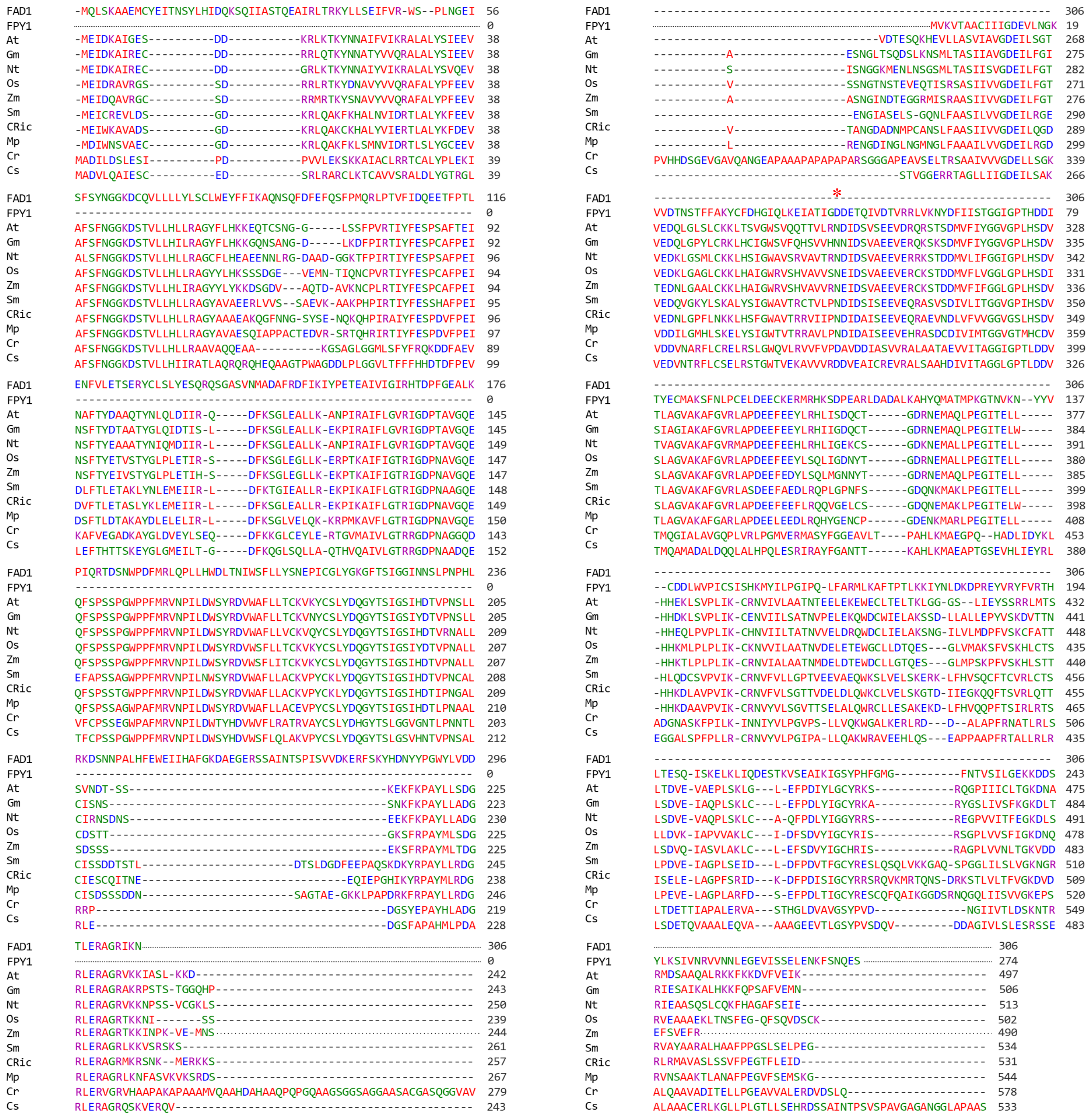

Figure S1: Multiple sequence alignment of plant FADS1 proteins. Alignments were generated using Clustal Omega and visualized using default colors. Domains with homology to *S. cerevisiae* FAD1 (left) and FPY1 (right) are shown separately. Accession numbers are provided in experimental procedures. Abbreviations for source species: At, *Arabidopsis thaliana*; Gm, *Glycine max*; Nt, *Nicotiana tabacum*; Os, *Oryza sativa*; Zm, *Zea mays*; Sm, *Sphagnum magellanicum*; CRic, *Ceratopteris richardii*; Mp, *Marchantia polymorpha*; Cr, *Chlamydomonas reinhardtii* ; Cs, *Chlorella sorokiniana*. Key residue for pyrophosphatase activity is designated with a red asterisk

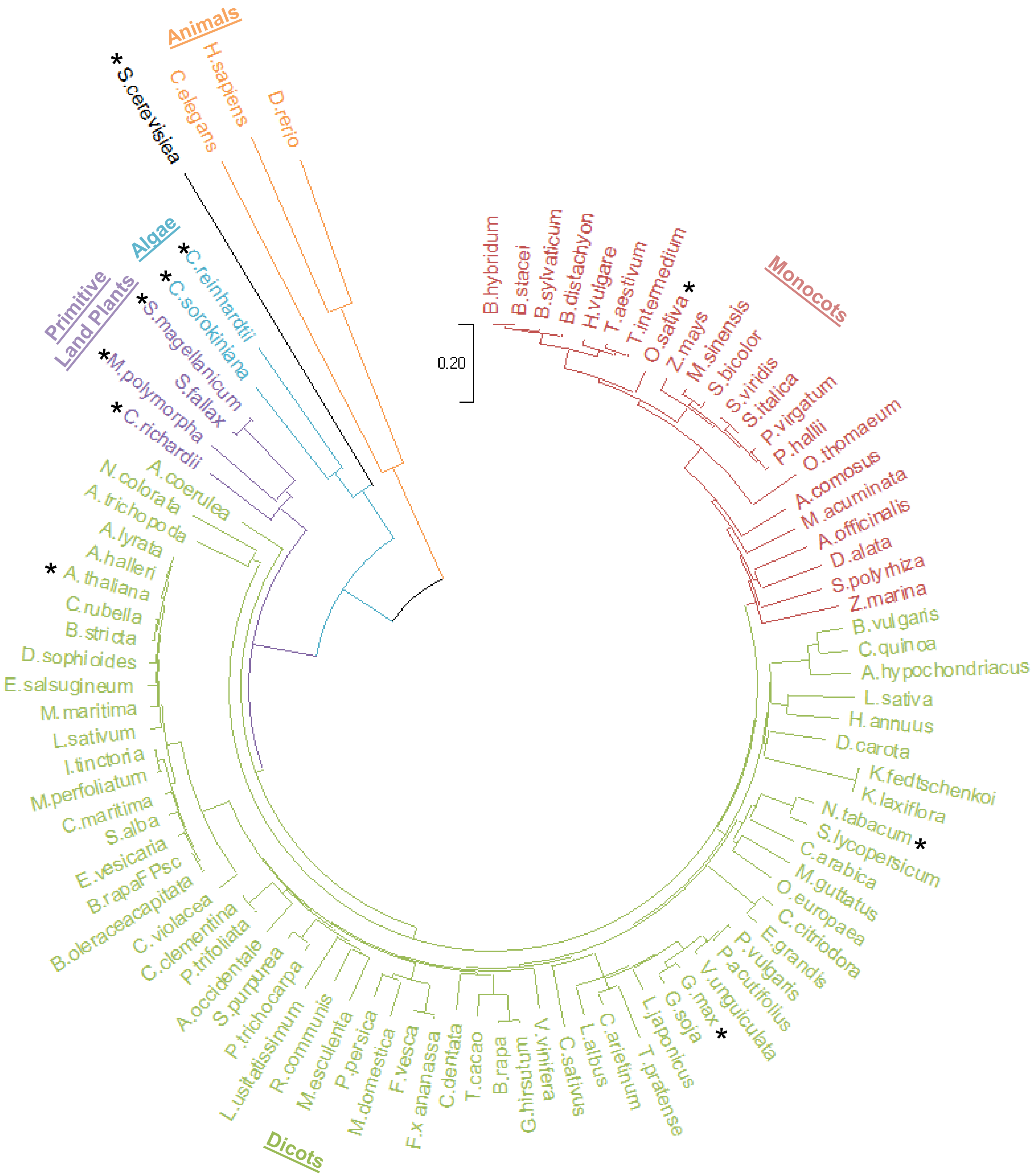

Figure S2: Phylogenetic analysis of FAD1/FPY1-like proteins. The full-length protein sequences of *S. cerevisiae* FPY1 and homologs across 93 plant and animal species were used to construct a neighbor-joining tree using MEGA 11. Asterisks denote sequences shown in Fig. S1.

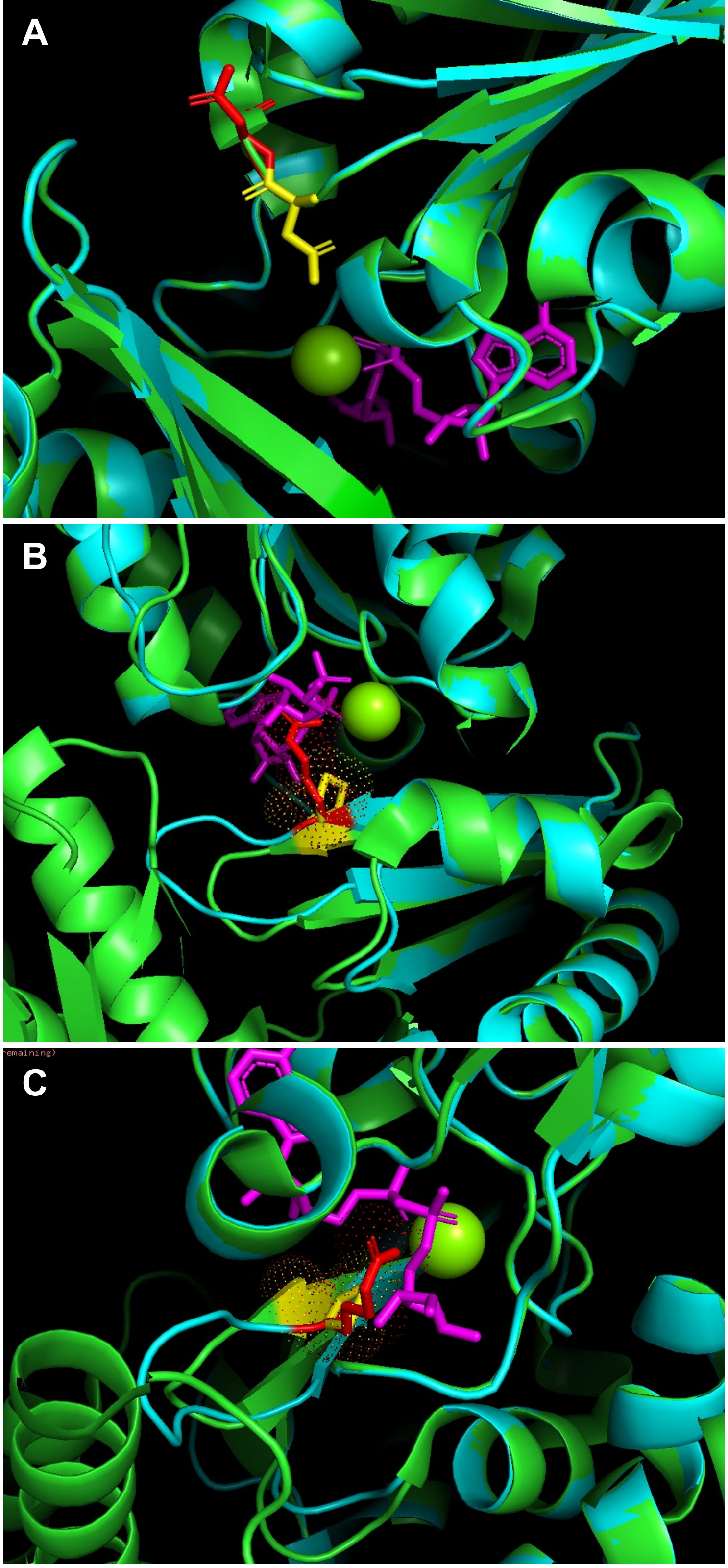

Figure S3: Homology-based modelling analysis of AtFADS1 pyrophosphatase active site. (A) The chain A pyrophosphatase active site of the *Thermus thermophilus protein* CinA protein (green), from a previous experimentally-determined crystal structure with bound substrate (magenta) and metal cation (green sphere), is overlaid with the modeled structure of AtFADS1 (blue). AtFADS1 N296, which occupies the same space as the metal-coordinating D45 in CinA, is shown in yellow. AtFADS1 D297 is shown in red. (B) The same overlayed structures are shown as in (A). CinA P210, including side chain, is shown in yellow, and the corresponding residue in AtFADS1, R458, is shown in red. (C) Different view of the same overlay shown in (B).

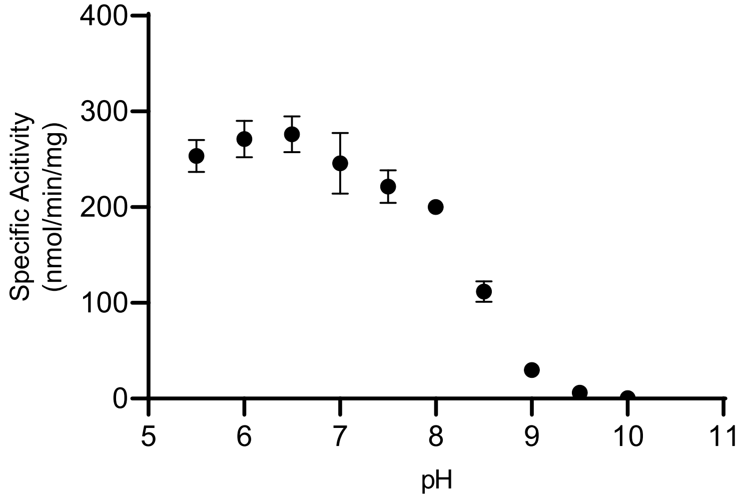

Figure S4: Effect of pH on FAD synthetase activity of AtFADS1. FAD synthetase activity was measured as described in Experimental Procedures, except reaction buffer was composed of 100 mM each MES, HEPES, and CHES to give a wide range of buffering capability. Data are means ± SEM of three replications.

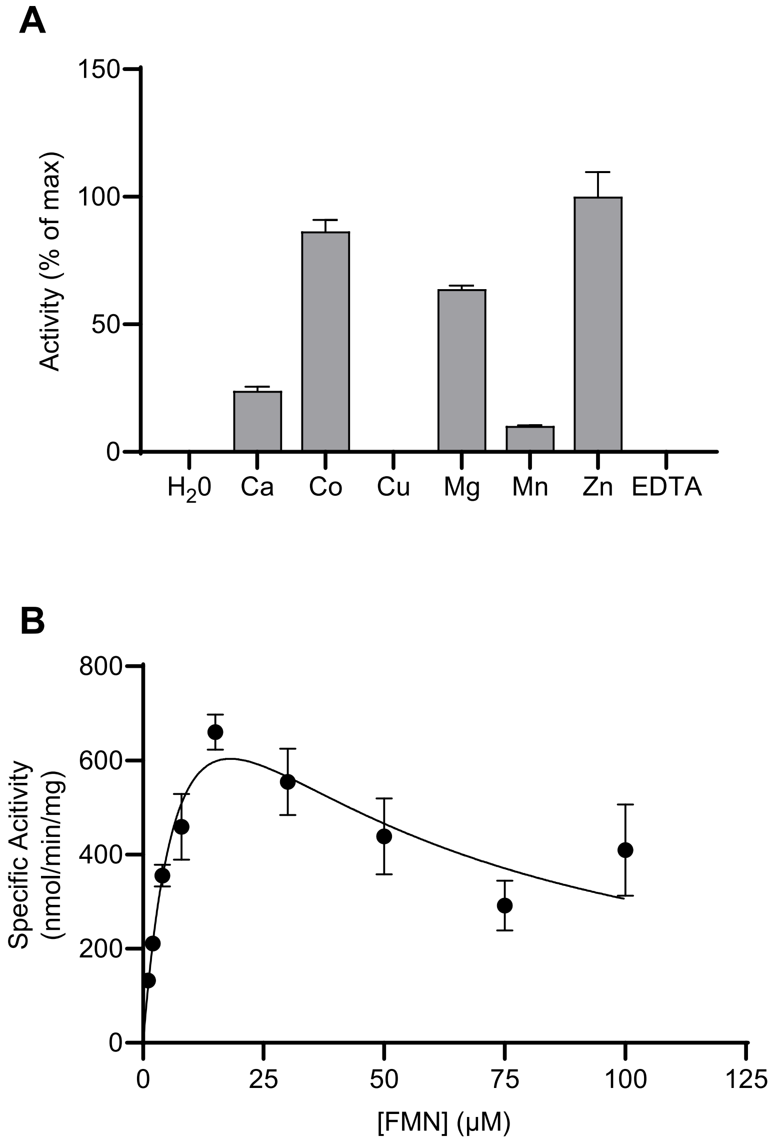

Figure S5: FAD synthetase activity of truncated AtFADS1^{trunc}. (A) Recombinant AtFAD^{trunc}, which lacks the FPY1-like domain, was assayed for FAD synthetase activity using 15 μM FMN, 10 mM ATP, and 10 mM salt of the divalent metal cations. Metal-free controls were performed using water or 2 mM EDTA in place of the ions. Data are expressed relative to the condition with maximum activity, set to 100%. (B) Initial reaction rates were determined using variable FMN concentrations with ATP concentration fixed at 10 mM. Curves represent a nonlinear best fit to a model of substrate inhibition. All data are means ± SEM of three triplicate determinations.

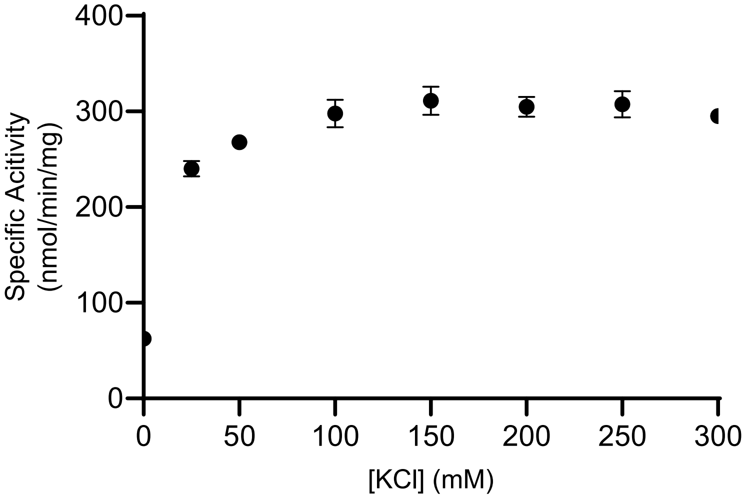

Figure S6: Potassium stimulation of the FAD pyrophosphatase activity of CrFADS1. Recombinant CrFADS1 was assayed for FAD pyrophosphatase activity as described in Experimental Procedures, except potassium chloride concentration was varied as shown. Data are means ± SEM of three triplicate determinations.

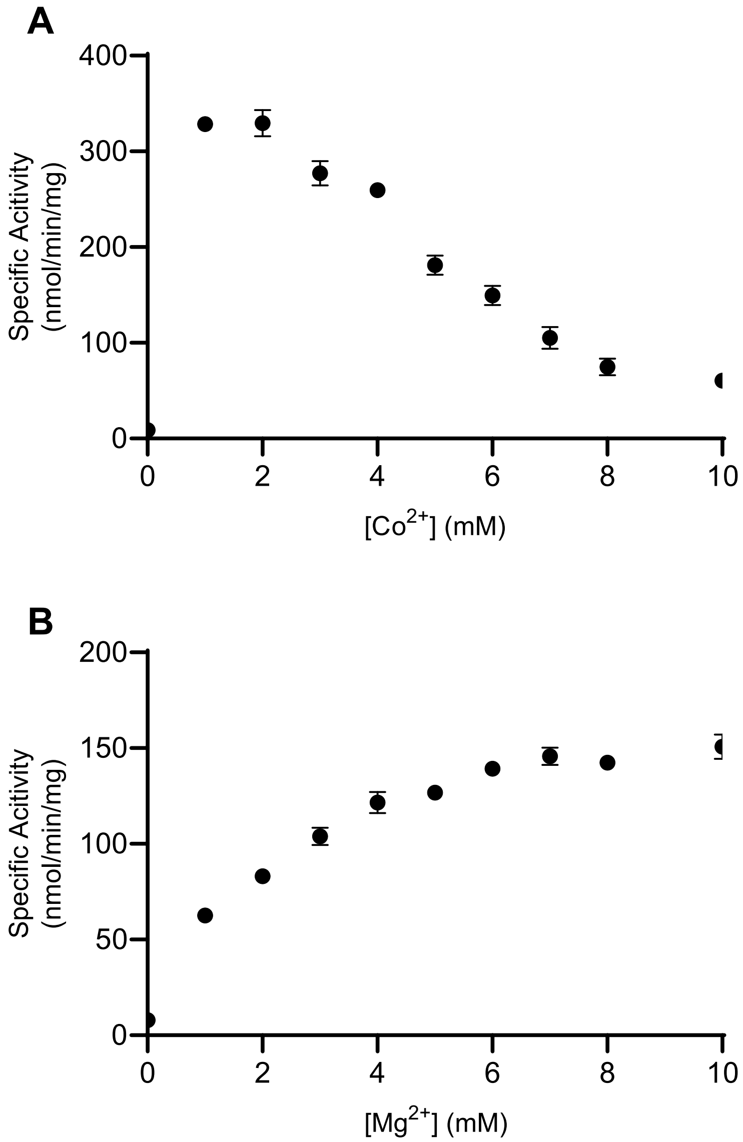

Figure S7: Metal dependence of the FAD pyrophosphatase activity of CrFADS1. Recombinant CrFADS1 was assayed for FAD pyrophosphatase activity in the presence of increasing concentrations of either (A) $CoCl_2$, or (B) $MgCl_2$. All other conditions were as described in experimental procures. Data are means ± SEM of three triplicate determinations.

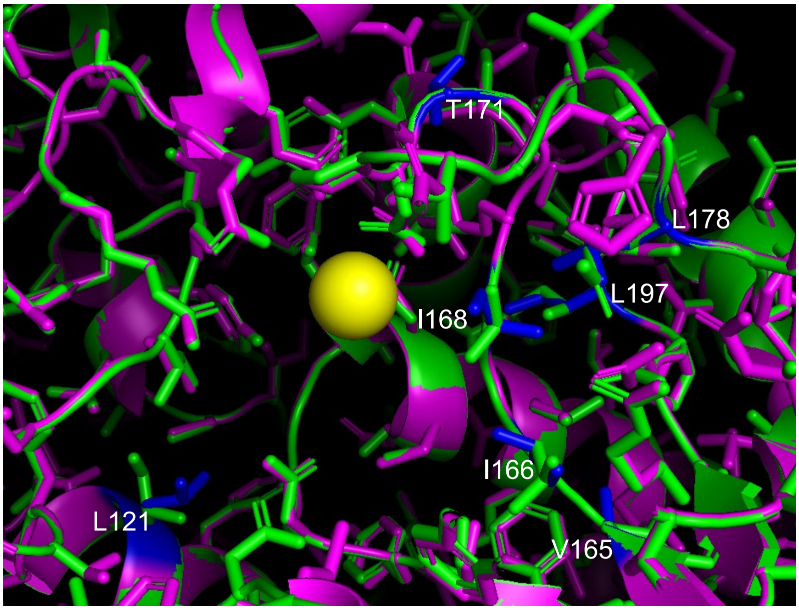

Figure S8: Homology-based modelling analysis of AtFADS1 FAD synthetase metal-binding site. The structure of AtFADS1 FAD synthetase domain (green) was inferred from homology-based modeling using, and overlaid with, the experimentally determined structure of *Candida glabrata* CgFMNAT (magenta) in complex with Mg^{2+} (yellow sphere). CgFMNAT residues that define the metal-binding pocket but are not conserved in AtFADS1 are shown in blue and labeled (CgFMNAT numbering).
